# IL-6–targeted therapies to block the cytokine or its receptor drive distinct alterations in T cell function

**DOI:** 10.1172/jci.insight.159436

**Published:** 2022-11-22

**Authors:** Cate Speake, Tania Habib, Katharina Lambert, Christian Hundhausen, Sandra Lord, Matthew J. Dufort, Samuel O. Skinner, Alex Hu, MacKenzie Kinsman, Britta E. Jones, Megan D. Maerz, Megan Tatum, Anne M. Hocking, Gerald T. Nepom, Carla J. Greenbaum, Jane H. Buckner

**Affiliations:** 1 Center for Interventional Immunology,; 2 Center for Translational Immunology,; 3 Center for Systems Immunology, and; 4 Immune Tolerance Network, Benaroya Research Institute at Virginia Mason, Seattle, Washington, USA.

**Keywords:** Autoimmunity, Immunology, Cytokines, Immunotherapy, T cells

## Abstract

Therapeutics that inhibit IL-6 at different points in its signaling pathway are in clinical use, yet whether the immunological effects of these interventions differ based on their molecular target is unknown. We performed short-term interventions in individuals with type 1 diabetes using anti–IL-6 (siltuximab) or anti–IL-6 receptor (IL-6R; tocilizumab) therapies and investigated the impact of this in vivo blockade on T cell fate and function. Immune outcomes were influenced by the target of the therapeutic intervention (IL-6 versus IL-6R) and by peak drug concentration. Tocilizumab reduced ICOS expression on T follicular helper cell populations and T cell receptor–driven (TCR-driven) STAT3 phosphorylation. Siltuximab reversed resistance to Treg-mediated suppression and increased TCR-driven phosphorylated STAT3 and production of IL-10, IL-21, and IL-27 by T effectors. Together, these findings indicate that the context of IL-6 blockade in vivo drives distinct T cell–intrinsic changes that may influence therapeutic outcomes.

## Graphical abstract

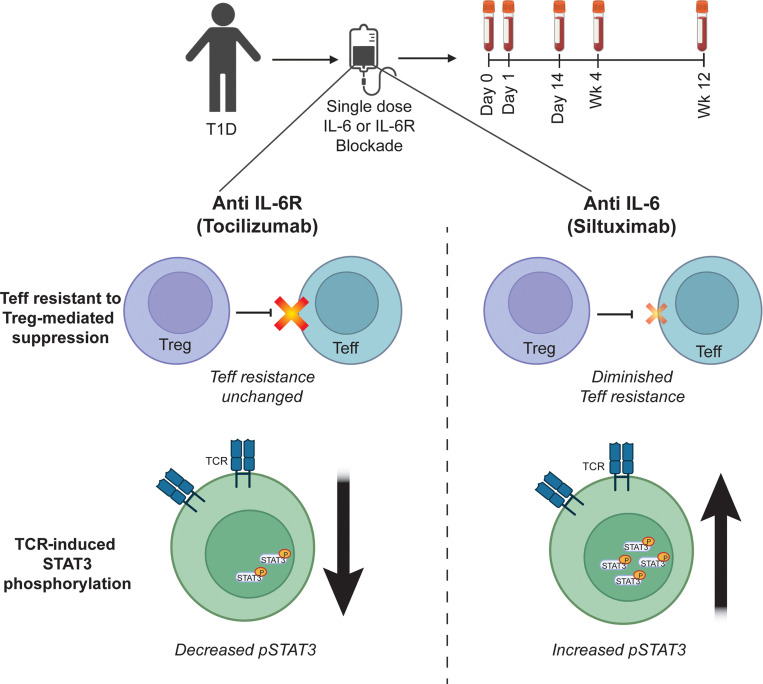

## Introduction

IL-6 is a pleiotropic cytokine important in the immune system, where it contributes to both innate and adaptive immune responses (1, 2). Overproduction of IL-6 and dysregulation of the IL-6 receptor (IL-6R) have been implicated in the pathogenesis of inflammatory disease, autoimmune disease, and cancer, driving the development of multiple drugs to block the IL-6 signaling pathway (2, 3). IL-6 signaling pathway inhibitors are currently approved to treat rheumatoid arthritis (RA); juvenile idiopathic arthritis; Castleman’s disease; giant cell arteritis; Takayasu arteritis; chimeric antigen receptor T cell–complicated cytokine release syndrome; and more recently, patients hospitalized with COVID-19 (1–4). Ongoing clinical trials are testing the safety and efficacy of IL-6 signaling pathway blockade in a growing list of other diseases (2, 5, 6). Currently, multiple approaches are being used to block the IL-6 signaling pathway therapeutically, including targeting IL-6 itself; its receptors IL-6R and GP130; and downstream effectors of IL-6 signaling, such as JAK and STAT3 (1, 3, 5).

Despite the therapeutic success of IL-6 signaling pathway blockade, less is known about the immune ramifications of these drugs in vivo, and it remains to be determined whether targeting different molecules in the pathway has differential effects on the immune cell subsets responsive to IL-6. To address this gap in knowledge, we used samples from 2 independent mechanistic clinical studies in patients with established type 1 diabetes (T1D) to compare the effect of anti–IL-6 (siltuximab) and anti–IL-6R (tocilizumab) therapies on T cell fate and function. We chose to focus on T cells based on numerous studies implicating IL-6 signaling in T cell dysregulation in T1D. IL-6 has been implicated in Th17 frequency and function in T1D (7, 8), and modifications in Treg frequency and function may also be attributable in part to IL-6 (9, 10). We and others have also shown that effector T cells (Teffs) from individuals with T1D are resistant to suppression by Tregs (11–13), and this Teff resistance appears to be STAT3 dependent (13). Furthermore, we have demonstrated that the T cell response to IL-6 signaling is enhanced in T1D in part because of increased IL-6R (14), although this has not been seen in all T1D cohorts (13). Despite these translational studies, the spectrum of T cell changes that are induced in humans in vivo by targeting the IL-6 pathway in individuals with autoimmunity is not well understood.

Here, we conducted 2 open-label, single-dose studies, which enabled us to investigate the in vivo impact of blocking IL-6 versus blocking IL-6R. These studies were conducted in individuals with T1D for 2 key reasons. First, as noted above, there is well-described dysregulation in the IL-6 pathway in T1D (7–14), and thus eventual possibility for utility in this patient population. Second, because standard of care in T1D does not yet involve immune-modulating drugs, these mechanistic studies do not affect the routine course of treatment for study participants by requiring them to change treatments or delay immunotherapy as they might in the setting of other autoimmune diseases. We evaluated the effect of each drug on T cell phenotype, response to IL-6, and Treg-mediated suppression and activation. We identified differential immune outcomes, highlighting the importance of understanding the in vivo impact of these therapies, which may not be detectable via in vitro studies. We propose that mechanistic trials like these could be used to evaluate other drug classes of interest, helping to explain drug mechanism of action and improving our understanding of human immunology more broadly.

## Results

### Study approach and pharmacokinetics.

In 2 independent single-arm, open-label clinical studies, a single dose of either anti–IL-6 (siltuximab) or anti–IL-6R (tocilizumab) therapy was administered to individuals with established T1D. Both studies were designed to assess predetermined mechanistic immunological endpoints, not clinical outcomes. There were 10 individuals in the siltuximab study and 9 individuals in the tocilizumab study. Importantly, 5 individuals participated in both studies, allowing an assessment of intraindividual responses to the 2 therapies; these individuals participated first in the siltuximab study and then in the tocilizumab study, and the time between administration of each drug ranged from 166 to 370 days. Blood samples were collected at 6 visits: screening at 2 weeks before infusion; a baseline visit with intravenous administration of the drug; and 4 follow-up visits at day 1, day 14, week 4, and week 12 after infusion. There were no significant differences between screening and day 0 for any of the assays, so for simplicity, only data for day 0 are presented. Given limited sample volumes, follow-up assays were not conducted on every individual or at all time points. Table 1 summarizes demographics, clinical characteristics, and serum drug concentrations. The individual patients and assays performed for each patient are listed in Supplemental Table 1 (supplemental material available online with this article; https://doi.org/10.1172/jci.insight.159436DS1). Supplemental Table 1 also lists an additional 3 patients treated with tocilizumab enrolled after completion of the primary cohort; samples from these individuals were used for secondary endpoint assays.

The half-lives of siltuximab and tocilizumab are 20.6 and 11 days, respectively. Peak drug concentration for both was measured at day 1 after infusion (Table 1). Neutrophil counts were monitored throughout the period of the study because neutrophil decline was expected as a consequence of these therapies (15–18). The frequency, magnitude, and kinetics of neutrophil decline differed considerably by drug (Supplemental Figure 1). At day 1 after siltuximab infusion, 5 individuals exhibited a slight decline in neutrophil counts, but none dropped below the normal range (Supplemental Figure 1A). However, at 28 days after infusion, 9 of the 10 individuals exhibited lower values compared with baseline. Of these 9 individuals, 5 showed clinically significant reductions, dropping below the lower limit of normal (LLN, 2 × 10^9^/μL); 3 had grade 1 neutropenia (1.5 × 10^9^/μL–2 × 10^9^/μL), 1 had grade 2 neutropenia (1.0 × 10^9^/μL–1.5 × 10^9^/μL), and 1 had grade 4 neutropenia (0 × 10^9^/μL–0.5 × 10^9^/μL). In contrast, both the magnitude and kinetics of neutrophil decline differed for tocilizumab. At day 1 after tocilizumab infusion, neutrophil levels declined for all individuals, with 5 individuals dropping below LLN; of these 5 individuals, 2 had grade 2 neutropenia, 2 had grade 3 neutropenia (0.5 × 10^9^/μL–1.0 × 10^9^/μL), and 1 had grade 4 neutropenia (Supplemental Figure 1B). Neutrophil count decline on day 1 after tocilizumab was modestly correlated with peak drug concentration (Pearson correlation, *r* = –0.66, *P* = 0.053). No infections or other adverse events occurred contemporaneously with low neutrophil counts in either study, and neutrophil counts normalized without intervention in all individuals by week 12.

### Comparison of IL-6–induced phosphorylated STAT3 in PBMCs isolated from tocilizumab-treated patients and siltuximab-treated patients.

**Figure 1:**
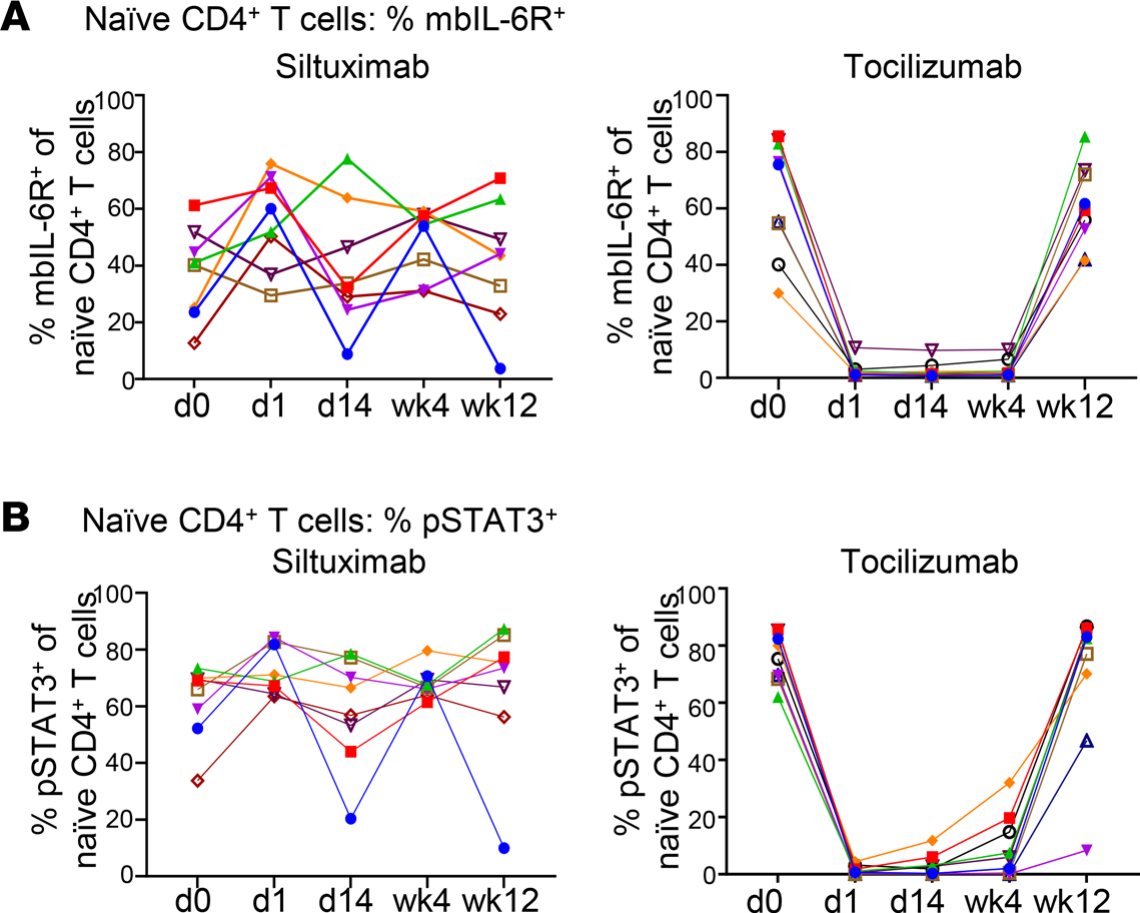
Suppression of IL-6–induced p-STAT3 persists in tocilizumab-treated but not siltuximab-treated patient PBMCs. Each line represents an individual patient; *n* = 10 for siltuximab and *n* = 9 for tocilizumab. (**A**) Frequency of mbIL-6R^+^ cells in naive CD4^+^ T cell compartment at baseline. (**B**) Frequency of IL-6–induced p-STAT3^+^ cells in the naive CD4^+^ T cell compartment. Thawed and rested PBMCs from siltuximab-treated or tocilizumab-treated patients with T1D were stimulated with recombinant IL-6 (2 ng/mL) for 10 minutes.

We first examined how each drug influences IL-6 signaling on T cells by measuring both membrane-bound IL-6R (mbIL-6R) expression and IL-6–induced phosphorylation of STAT3 in cryopreserved PBMCs from individuals treated with each drug. After siltuximab treatment, we found no change in IL-6R expression on the cell surface of naive CD4^+^ T cells (Figure 1A and Supplemental Figure 2A); also, there was no change in soluble IL-6R serum levels (Supplemental Figure 2B). In contrast, after tocilizumab treatment, we detected less IL-6R on the cell surface of naive CD4^+^ T cells at day 1, day 14, and week 4 with a return to preinfusion levels by week 12 (Figure 1A). Since this loss of detection may be due to tocilizumab bound to the mbIL-6R and blocking binding of the anti–IL-6R antibody, we tested side by side 2 monoclonal anti–IL-6R antibodies: clone UV4, which was used in Figure 1A, and clone M5. In this experiment, PBMCs from a healthy control individual were preincubated in the presence or absence of tocilizumab and then stained with UV4 or M5. In the absence of tocilizumab, both clones were able to detect cell-surface IL-6R, although M5 staining was more robust than UV4 (Supplemental Figure 3A, left). However, in the presence of tocilizumab, only M5 but not UV4 was able to bind and hence detect IL-6R (Supplemental Figure 3A), indicating that M5 binds to a different epitope on mbIL-6R than tocilizumab, whereas UV4 competes with tocilizumab binding to mbIL-6R. Therefore, the reduction of mbIL-6R levels shown in Figure 1A was likely due to the presence of tocilizumab bound to mbIL-6R, thus preventing binding of the UV4 monoclonal antibody. These results also suggest that after a single dose, tocilizumab was bound to mbIL-6R on T cells for at least 4 weeks after treatment but was no longer present at week 12 (Figure 1A). To confirm that M5 binds to a different IL-6R epitope than tocilizumab, we stained with both M5 and fluorescently labeled tocilizumab and demonstrated dual staining (Supplemental Figure 3B). We then examined PBMCs still available from our in vivo study using the M5 monoclonal antibody to assess IL-6R expression in 3 patients treated with tocilizumab (Supplemental Figure 3, C and D). With this new antibody, we found no change in mbIL-6R levels after tocilizumab, yet we did see a change in phosphorylated STAT3 (p-STAT3) consistent with tocilizumab binding at week 2 (Supplemental Figure 3, C and D). Together, these results suggest that although tocilizumab remained bound to mbIL-6R on CD4^+^ T cells for at least 4 weeks after the single-dose treatment, it did not alter the levels of mbIL-6R over 12 weeks.

Siltuximab and tocilizumab had differential effects on the frequency of IL-6–induced p-STAT3^+^ naive CD4^+^ T cells isolated from PBMCs from treated individuals. There was no change in the frequency of IL-6–induced p-STAT3^+^ naive CD4^+^ T cells after siltuximab; this may be explained by siltuximab being washed out when PBMCs are isolated. In contrast, there was a substantial reduction after tocilizumab (Figure 1B, Supplemental Figure 3F and Supplemental Figure 4), which was rapid and almost complete at day 1 after tocilizumab infusion, consistent with the persistent binding of tocilizumab to the mbIL-6R on PBMCs. Of note, there was heterogeneity in the rate of return to preinfusion levels among tocilizumab-treated patients, where some individuals began to return to preinfusion levels at week 2 while other individuals still had significantly reduced levels at week 12 compared with day 0 levels (Figure 1B). Moreover, this heterogeneity did not correlate with peak drug concentration or with most proximal drug concentration at 4 weeks (data not shown); no serum tocilizumab was detectable for any individual by 12 weeks after infusion. Further investigation demonstrated that tocilizumab but not siltuximab reduced IL-6–induced p-STAT3 in other T cell subsets, including memory CD4^+^ T cells, memory CD8^+^ T cells, and CD4^+^ Tregs (Supplemental Figure 5) with similar kinetics and individual-by-individual heterogeneity in rate of return to responsiveness as the naive CD4^+^ T cell population. Likewise, IL-6–induced p-STAT1 was strongly modified after tocilizumab, not siltuximab, in in vitro assays of each of these populations (Supplemental Figure 6). Together, these findings indicate that tocilizumab achieved global suppression of IL-6–induced p-STAT3 signaling in T cells within 24 hours after a single dose, which was maintained after PBMC isolation due to persistent binding of tocilizumab to the mbIL-6R for at least 4 weeks after infusion.

### Tocilizumab but not siltuximab decreases ICOS expression on T follicular helper cells.

**Figure 2:**
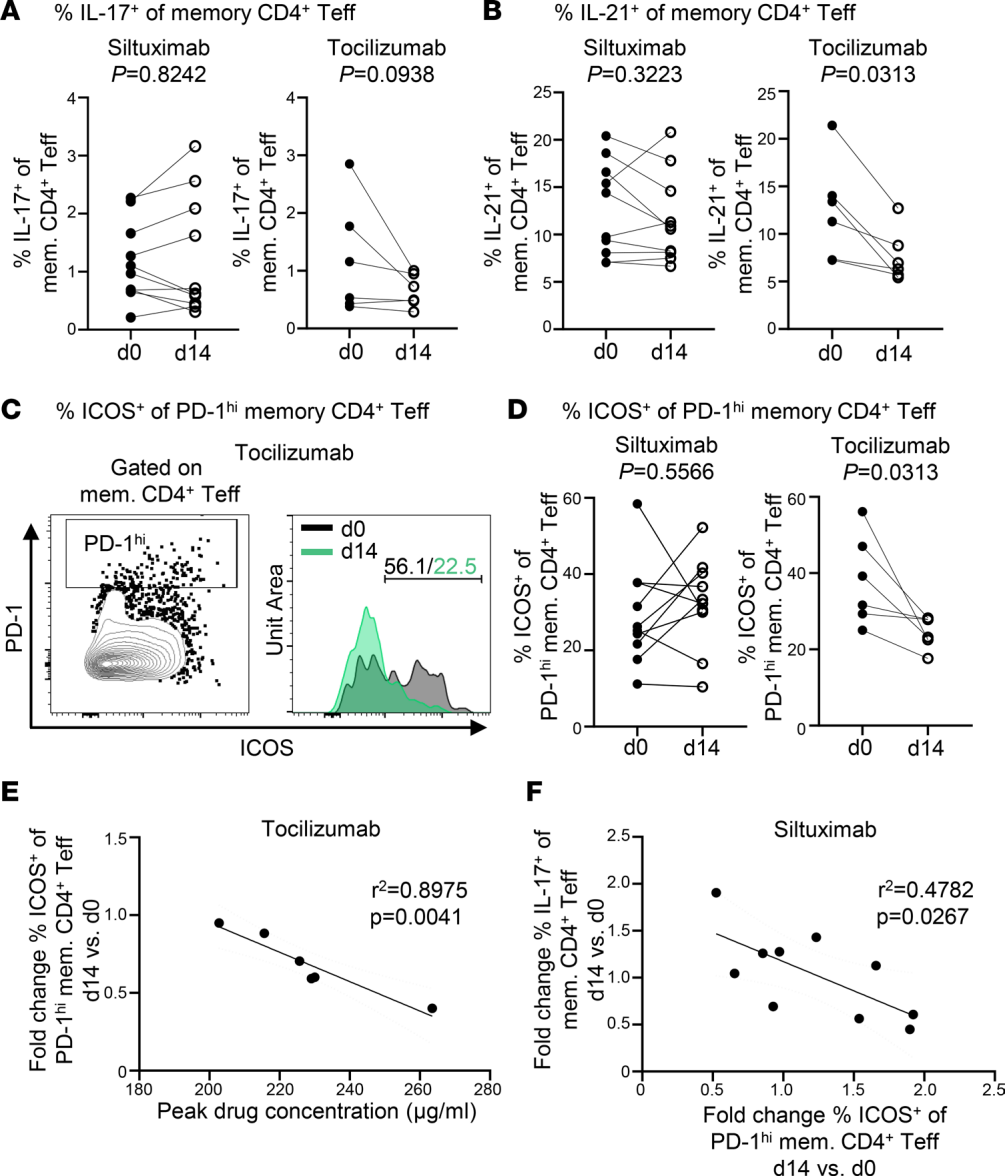
Tocilizumab but not siltuximab decreases ICOS expression of T follicular helper cells. Thawed and rested PBMCs from siltuximab-treated or tocilizumab-treated patients with T1D were stimulated with PMA/ionomycin for 1 hour followed by an additional 3 hours in the presence of Brefeldin A. Each line represents an individual patient; *n* = 10 for siltuximab and *n* = 6 for tocilizumab. Solid circles represent d0 prior to drug infusion, and open circles represent d14 after drug infusion. (**A**) Frequency of IL-17^+^ cells in memory CD4^+^ Teffs. (**B**) Frequency of IL-21^+^ cells in memory CD4^+^ Teffs. (**C**) Representative histograms showing PD-1^hi^ ICOS^+^ memory CD4^+^ Teffs at d0 and d14 after tocilizumab infusion from a single patient. (**D**) Frequency of ICOS^+^ cells in PD-1^hi^ memory CD4^+^ Teffs. (**E**) Linear regression for tocilizumab cohort showing negative correlation between peak drug concentration on d1 and fold change d14 versus d0 for frequency of ICOS^+^ cells in PD-1^hi^ memory CD4^+^ Teffs. (**F**) Linear regression for siltuximab cohort showing negative correlation between fold change d14 versus d0 for frequency of ICOS^+^ cells in PD-1^hi^ memory CD4^+^ T cell compartment and fold change d14 versus d0 for frequency of IL-17^+^ cells in memory CD4^+^ T cell compartment. Statistical tests: (**A**, **B**, and **D**) Wilcoxon matched-pairs signed-rank test; and (**E** and **F**) linear regression.

We next investigated the impact of both drugs on T cell subsets for which IL-6 signaling is known to play a role in determining phenotype. These include follicular T (Tfh) cells, peripheral T helper (Tph) cells, Th17 cells, and Tregs (19–24). We first assessed the frequency of CD4^+^ and CD8^+^ naive and memory T cells, CD4^+^ Tregs (CD4^+^CD25^hi^CD127^lo^), and Th17 cells (CD4^+^FOXP3^–^IL-17^+^) at each time point and found no change in frequency in response to either drug for any of these populations (data not shown, Supplemental Figure 7, A and B). We also confirmed that there was no change to FOXP3 MFI on Tregs (Supplemental Figure 7C). We performed a more comprehensive assessment of T cell subsets, analyzing PBMCs from days 0 and 14, and observed that siltuximab treatment had either no effect or resulted in an increased frequency of IL-17^+^ memory CD4^+^ Teffs (Figure 2A). In contrast, although not statistically significant, tocilizumab resulted in a decreased frequency of IL-17^+^ memory CD4^+^ Teffs for 5 of 6 individuals tested (Figure 2A), suggesting that a single dose of tocilizumab can impact Th17 function or lineage development. Additionally, intracellular cytokine staining after PMA/ionomycin stimulation showed that tocilizumab reduced the frequency of memory CD4^+^ Teffs capable of producing IL-21 (Figure 2B) as well as IL-21^+^FOXP3^+^ Tregs (Supplemental Figure 8A). This suggests that tocilizumab but not siltuximab may impair Tfh and/or Tph differentiation or function, both of which produce IL-21 (25). To address this further, high expression of PD-1 was used as a marker of both Tfh and Tph; however, we were unable to differentiate Tfh from Tph because of a failure of CXCR5 in our panel. After tocilizumab treatment, the frequency of PD-1^hi+^ memory CD4^+^ Teffs was lower in 4 of the 6 individuals (Supplemental Figure 8B), whereas there was no consistent pattern for frequency of PD-1^hi+^ memory CD4^+^ Teffs in the siltuximab-treated patients (Supplemental Figure 8B). Since IL-6R expression has been shown to be required for Tfh expression of ICOS (24), we examined ICOS expression on PD-1^hi+^ memory CD4^+^ Teffs. We found that in response to tocilizumab but not siltuximab, there was a significant decrease in ICOS expression among PD-1^hi+^ memory CD4^+^ Teffs (Figure 2, C and D), and this decrease in ICOS expression correlated significantly with the peak drug concentration measured at day 1 after drug infusion (Figure 2E). ICOS expression was modestly but not significantly decreased among FOXP3^+^ Tregs after tocilizumab, while overall the frequency of FOXP3^+^ Tregs remained unchanged (Supplemental Figure 8, C and D). To confirm that siltuximab did not modify the homeostatic relationship between these cell populations, we next assessed the correlations between fold change in frequency for ICOS^+^ PD-1^hi^ memory CD4^+^ Teffs, IL-17^+^ memory CD4^+^ Teffs, and FOXP3^+^ Tregs after siltuximab exposure. There was an inverse correlation between ICOS^+^PD^–^1^hi^ memory CD4^+^ Teffs and IL-17^+^ memory CD4^+^ Teffs (Figure 2F). Additionally, FOXP3^+^ Tregs correlated inversely with ICOS^+^PD-1^hi+^ memory CD4^+^ Teffs (Supplemental Figure 8E) but correlated positively with IL-17^+^ memory CD4^+^ Teffs (Supplemental Figure 8F). Collectively, these findings suggest that direct blockade of IL-6 signaling with tocilizumab had a greater impact on Th17 and Tfh/Tph lineages and ICOS expression than siltuximab.

### Siltuximab but not tocilizumab reverses Teff resistance to Treg-mediated suppression.

**Figure 3:**
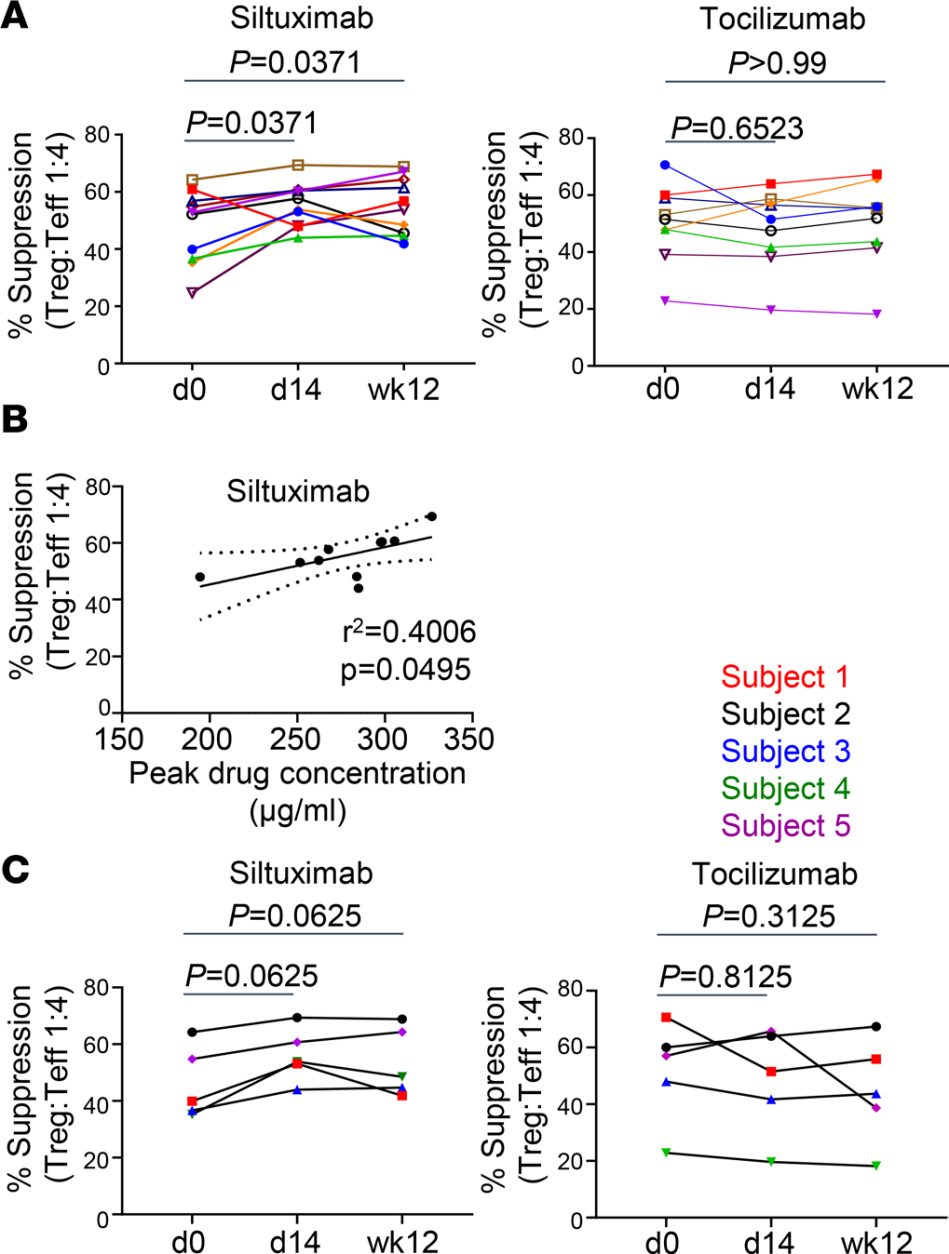
Siltuximab but not tocilizumab reverses Teff resistance to Treg-mediated suppression. Expanded allogeneic Tregs from a healthy control donor were cocultured at a ratio of 1:4 with Teffs from either siltuximab-treated or tocilizumab-treated patients with T1D in the presence of anti-CD3/anti-CD28–coated beads for 2 days. The percentage of suppression was determined by measuring the frequency of activated CD25^+^CD134^+^ Teffs. Each line represents an individual patient; *n* = 10 for siltuximab and *n* = 9 for tocilizumab. (**A**) Percentage suppression for siltuximab-treated patients and tocilizumab-treated patients. (**B**) Linear regression for siltuximab cohort showing positive correlation between peak drug concentration on d1 and percentage suppression at d14. (**C**) Percentage suppression for cohort that participated in both studies (*n* = 5); note these individuals are also included in **A** and **B**. Statistical tests: (**A** and **C**) Wilcoxon matched-pairs signed-rank test; (**B**) linear regression.

We and others have previously shown that CD4^+^ Teffs are resistant to Treg suppression in established T1D (11–13). IL-6 has been implicated in the resistance of T cells to Treg-mediated suppression (13, 26–28). To determine whether Teff resistance to Treg suppression could be modified in vivo by either drug, we used an in vitro suppression assay (29) to assess the suppression of Teffs isolated from PBMCs at day 0, day 14, and week 12 by a standard pool of Tregs derived from a healthy control individual. Strikingly, siltuximab but not tocilizumab enhanced suppression of Teffs by Tregs (Figure 3). At day 14 after siltuximab treatment, Teff resistance was reversed in 9 out of 10 individuals (Figure 3A). These improved levels of response to Treg suppression were sustained to 12 weeks after infusion in 5 of the 9 individuals (Figure 3A). Notably, the percentage of suppression levels at day 14 correlated with peak drug concentration at day 1 (Figure 3B). This was not seen after tocilizumab, where there was no change in resistance to suppression at either day 14 or week 12 after drug infusion (Figure 3A). Markedly, these drug-specific differences were seen in the individuals who participated in both studies (Figure 3C). These findings suggest that the influence of these 2 IL-6–targeted therapies resulted in drug-specific alterations in response to activation that were T cell intrinsic and influenced their response to regulation. To address these differences, we interrogated the changes in the response to T cell receptor activation.

### Tocilizumab and siltuximab have opposing effects on TCR-induced p-STAT3 signaling.

**Figure 4:**
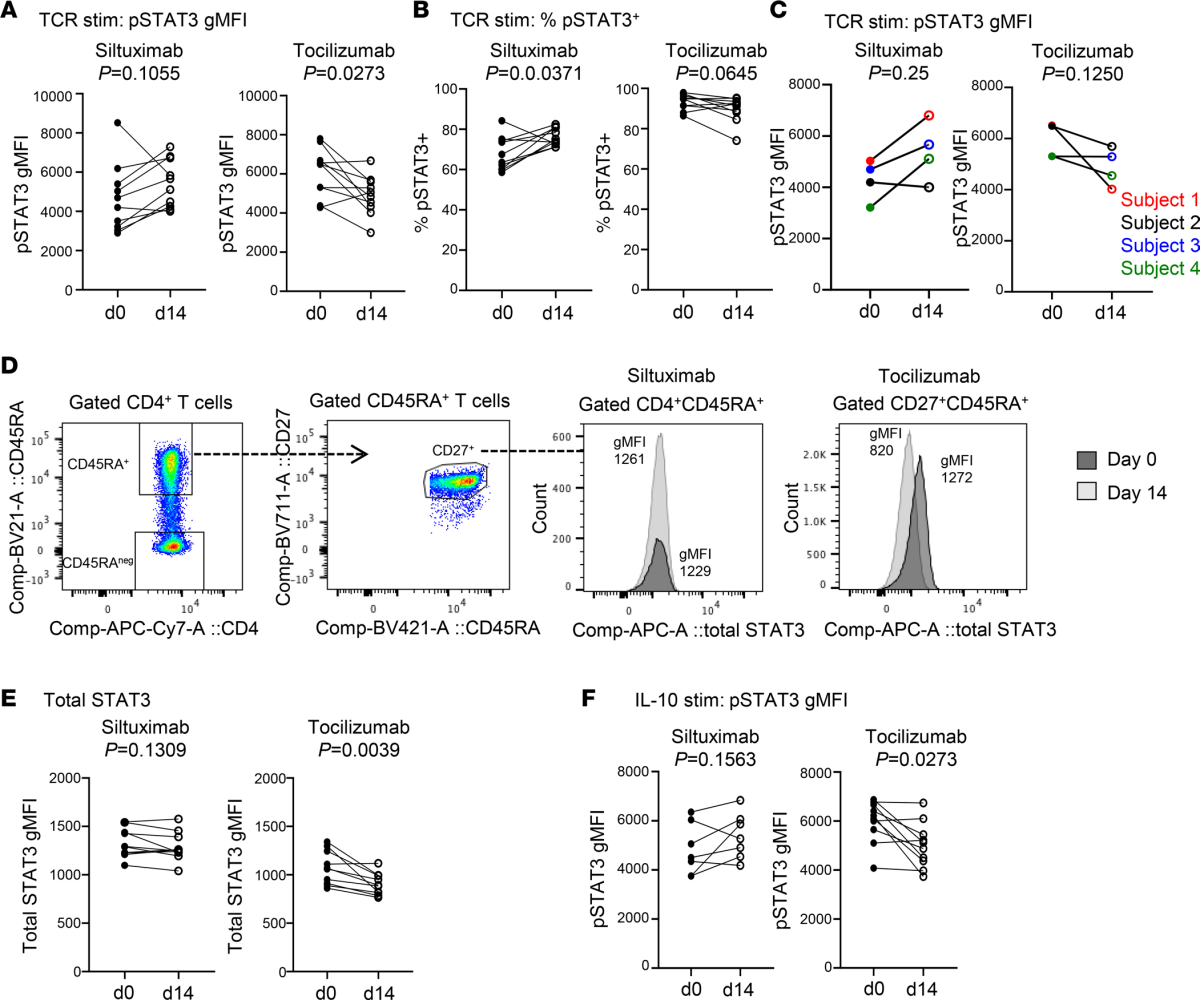
Tocilizumab and siltuximab have opposing effects on TCR-induced p-STAT3 signaling in naive CD4^+^ T cells. Enriched pan T cells from siltuximab-treated or tocilizumab-treated patients with T1D were stimulated or not with anti-CD3/anti-CD28–coated beads for 4 hours. Cells were stained for p-STAT3 and total STAT3. Each line represents an individual patient; *n* = 10 for siltuximab (except for **F** where *n* = 7; gated CD4^+^CD45RA^+^ naive CD4^+^ T cells) and *n* = 10 for tocilizumab (gated CD4^+^CD27^+^CD45RA^+^ naive CD4^+^ T cells). Solid circles represent d0 prior to administration of the drug, and open circles represent d14 after drug administration. (**A**) p-STAT3 geometric MFI (gMFI). (**B**) Frequency of p-STAT3^+^ cells. (**C**) p-STAT3 MFI for cohort that participated in both studies (*n* = 4); note these individuals are also included in **A**, **B**, **E**, and **F**. (**D**) Gating strategy for total STAT3 gMFI of gated naive CD4^+^ T cells using unstimulated enriched pan T cells: histograms for representative siltuximab-treated patient and representative tocilizumab-treated patient. (**E**) Total STAT3 MFI of unstimulated cells. (**F**) p-STAT3 MFI after stimulation with IL-10 (20 ng/mL for 30 minutes). Statistical tests: Wilcoxon matched-pairs signed-rank test.

TCR-mediated phosphorylation of STAT3 has previously been shown to be enhanced in CD4^+^ Teffs from patients with T1D compared with healthy control individuals, suggesting altered kinetics of STAT3 activation in patients with T1D (13). To determine the effect of tocilizumab or siltuximab on TCR-induced STAT3 activation at days 0 and 14, we stimulated enriched CD3^+^ T cells with anti-CD3/CD28 beads and measured STAT3 phosphorylation across CD4^+^ and CD8^+^ naive and memory T cell subsets (Supplemental Figure 9A), comparing day 0 and day 14. Strikingly, TCR-induced p-STAT3 MFI was increased in 8 of 10 individuals who received siltuximab but decreased in 7 of 10 individuals who received tocilizumab (Figure 4A). TCR-induced p-STAT3^+^ cell frequency was increased in 8 of 10 patients treated with siltuximab but decreased in 7 of 10 patients treated with tocilizumab (Figure 4B). Again, these drug-specific effects were seen in individuals participating in both studies (Figure 4C). Total STAT3 MFI was decreased in tocilizumab-treated patients but was not significantly changed in those treated with siltuximab (Figure 4, D and E). The opposing effects of tocilizumab and siltuximab on TCR-induced STAT3 activation was most prominent in naive CD4^+^ T cells but also observed in memory CD4^+^ T cells (Supplemental Figure 9, B–D) as well as in naive CD8^+^ T cells (Supplemental Figure 9, E–G). Further investigation showed that the alteration in p-STAT3 signaling in response to siltuximab appears to be specific to TCR stimulation, as there was no enhancement in IL-10–induced p-STAT3 MFI (Figure 4F). Conversely, IL-10–induced p-STAT3 MFI was decreased in tocilizumab-treated individuals (Figure 4F), consistent with the observed reduction in total STAT3 expression (Figure 4E), and indicating that the decrease was not due solely to blockade of IL-6R. We also assessed TCR-induced p-STAT1 and p-STAT5. Tocilizumab treatment resulted in a decrease in both TCR/p-STAT1 and TCR/p-STAT5, whereas no significant alterations in TCR/p-STAT1 or TCR/p-STAT5 were found in T cells from siltuximab-treated patients (Supplemental Figure 10). Tocilizumab also decreased total STAT5 levels but had no effect on total STAT1 (Supplemental Figure 10). Collectively, these findings suggest that the effect of tocilizumab on STAT activation after TCR stimulation was driven by a direct effect of treatment on total STAT expression, whereas the response to TCR stimulation after siltuximab treatment resulted in an alteration that was specific to STAT3 signaling.

### Siltuximab but not tocilizumab enhances T cell production of regulatory cytokines.

**Figure 5:**
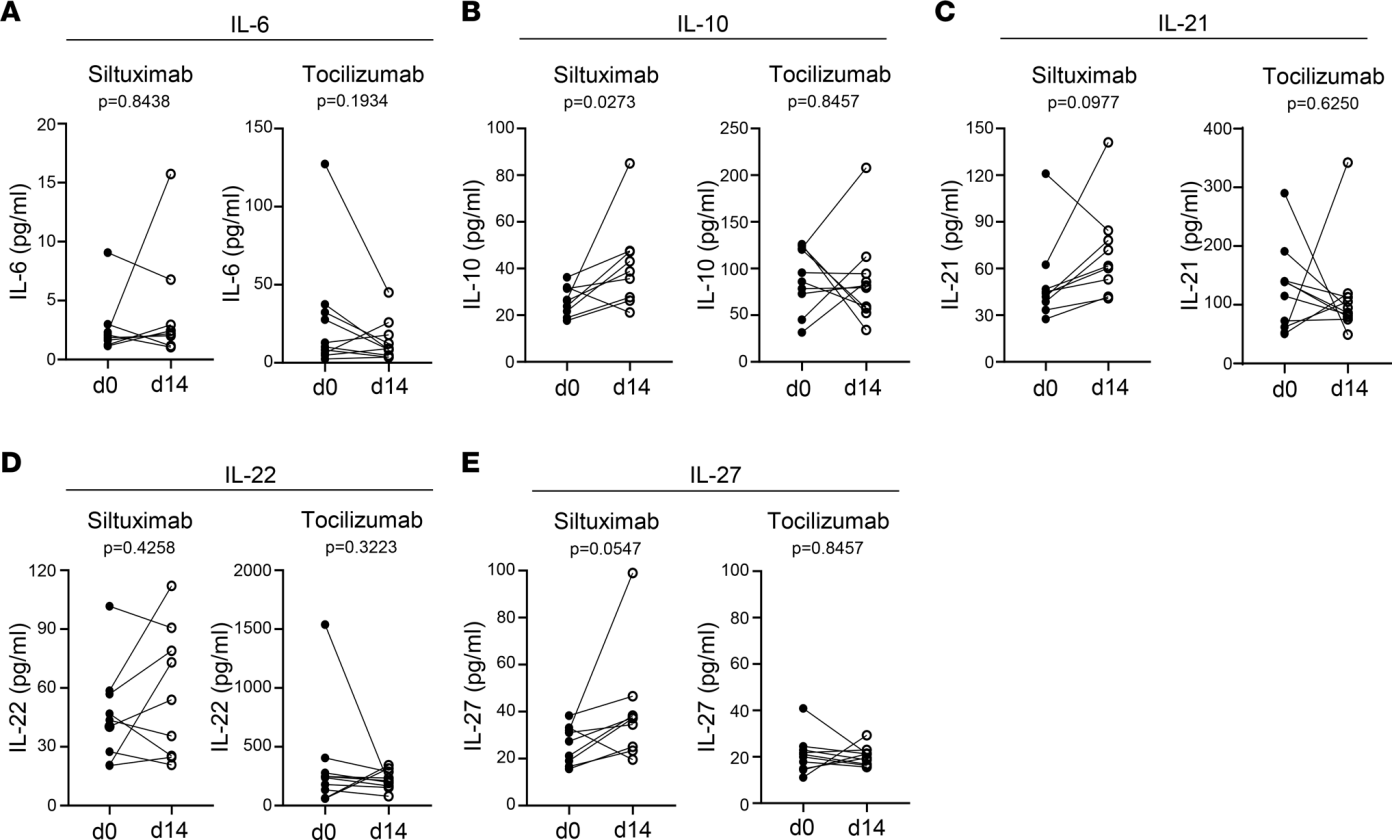
Siltuximab but not tocilizumab enhances T cell production of regulatory cytokines. Meso Scale Discovery assays were used to measure cytokine production by enriched pan T cells from siltuximab-treated or tocilizumab-treated patients with T1D stimulated with anti-CD3/anti-CD28–coated beads for 4 hours. Each line represents an individual patient; *n* = 9 for siltuximab (except for **A** where *n* = 8) and *n* = 10 for tocilizumab. Solid circles represent d0 prior to administration, and open circles represent d14 after drug administration (d14). (**A**) IL-6, (**B**) IL-10, (**C**) IL-21, (**D**) IL-22, and (**E**) IL-27. Statistical test: Wilcoxon matched-pairs signed-rank test.

To further explore the differential effects of siltuximab and tocilizumab on the response to TCR activation, we examined cytokine production using a multiplexed assay to measure IL-6, IL-10, IL-21, IL-22, and IL-27, since these cytokines have been reported to regulate both immunogenic and tolerogenic responses (30–32). Siltuximab treatment resulted in increased production of IL-10, IL-21, and IL-27 in 9 of 10 individuals but had no effect on IL-6 and IL-22 production (Figure 5). Conversely, tocilizumab had no effect on any of these cytokines (Figure 5). To further investigate the link between enhanced TCR-induced production of IL-10, IL-21, and IL-27 and the enhanced STAT3 phosphorylation observed in response to siltuximab, we calculated Pearson correlations between 4-hour cytokine production and frequency of p-STAT3^+^CD4^+^ T cells from day 0 and day 14. At day 14, TCR-induced p-STAT3 had a positive correlation with IL-10 (*R*^2^ = 0.4726, *P* = 0.04) and IL-27 (*R*^2^ = 0.4490, *P* = 0.0483) but a slightly weaker correlation for IL-21 (*R*^2^ = 0.3933, *P* = 0.07). There was no correlation at either time point between TCR-induced p-STAT3 and IL-22, an IL-10 family cytokine that shares the IL-10R2 receptor with IL-10 (33–35), demonstrating the specificity of our findings.

To exclude the possibility that altered cytokine receptor expression is associated with the enhanced TCR-induced p-STAT3 signaling observed in response to siltuximab, we assessed cell-surface levels of IL-10R, IL-21R, IL-27R, and gp130. Although limited by sample availability for this question, no differences in expression were observed between days 0 and 14 for any of these receptors (Supplemental Figure 11). Taken together, these findings further distinguish the impact of tocilizumab and siltuximab on T cell function in vivo, suggesting indirect effects of IL-6 blockade with siltuximab, as opposed to the direct effect of blunted IL-6R signaling seen with tocilizumab, which leads to enhanced regulatory cytokine expression and p-STAT3 responses.

## Discussion

In this study, we compared the impact of 2 therapies targeting the IL-6 pathway on T cell fate and function. Importantly, by using a single dose of each drug and sampling over time, we were able to assess how these 2 therapies have divergent effects. We propose that these differences are driven by distinct mechanisms of action in which tocilizumab directly blocks IL-6 signaling on T cells and siltuximab less directly blocks signaling by altering the cytokine milieu to which T cells are exposed.

Several results were expected. In tocilizumab-treated patients, the almost complete blockade of IL-6–induced p-STAT3 signaling in our in vitro stimulation assay and the lack of staining for cell-surface IL-6R was expected. This was due to persistent binding of tocilizumab to the IL-6R on the T cell surface, blocking in vitro activation upon addition of IL-6. In contrast, siltuximab was not present during our in vitro stimulation studies, as it would not persist through PBMC isolation, and thus did not alter the ability of T cells to respond to IL-6 in vitro. Changes in the frequency of T cell populations were modest, likely due to the short time period studied and the single dose of drug administered. In patients with RA, clear changes in Treg, Th17, and Tfh frequency have been documented after IL-6R blockade, albeit after months of therapy (20, 36, 37). However, it was noteworthy that a change in the percentage of T cells producing IL-17 and IL-21 could be identified with only 1 dose, suggesting that even a single dose has an impact on cytokine production and potentially the initiation of lineage commitment.

The difference in suppression of Teffs by Tregs seen with siltuximab compared with tocilizumab was unexpected. Multiple factors contribute to resistance to suppression by Tregs, including inflammatory cytokines TNF-α, IL-1, IL-21, and IL-6 as well as cell-intrinsic factors. Blockade of inflammatory cytokines has been shown to reverse Teff resistance, as is the case with anti-TNF therapy in RA (38). In T1D, in vitro studies indicate that resistance to Tregs is cell intrinsic (11). STAT3 has also been implicated in Teff resistance and the reversal of the resistant phenotype in autoimmunity with STAT3 inhibitors (26). Ihantola and colleagues linked enhanced CD2/CD3/CD28–p-STAT3 responses to Teff resistance in T1D (13). Yet, we observed decreased p-STAT3 after TCR/CD28 activation with tocilizumab, where no reversal of Teff resistance was seen. The decrease in p-STAT3 was not specific to the IL-6 response and was likely due in part to the decrease in total STAT expression, an outcome of the direct effect of IL-6R blockade on these cells (39, 40). It is possible that the tocilizumab-driven decrease in TCR-induced p-STAT3 was too marginal to effect a change in response to Tregs as compared with settings where STAT3 is completely inhibited (26). Notably, the lack of change in response to Tregs was not due to the short course of therapy, as we were unable to detect any change in suppression in participants in the EXTEND trial, a phase II clinical trial of tocilizumab in new onset T1D after 1 year of treatment (6).

In contrast, we saw a reversal in Teff resistance with siltuximab even though only p-STAT3 signaling, and not p-STAT1 or p-STAT5, was enhanced after T cell activation. This increase in TCR-induced p-STAT3 was likely due to secreted cytokines, and consistent with this was the increase in production of IL-10, IL-21, and IL-27 after T cell activation in the patients treated with siltuximab, a feature not seen with tocilizumab. All 3 cytokines may contribute to this increase in TCR-induced p-STAT3 after siltuximab therapy, but a significant correlation was only seen at day 14 with IL-10 and IL-27. Furthermore, it is notable that IL-10 only signals through STAT3, whereas IL-21 and IL-27 have the capacity to phosphorylate STAT1 and STAT5, which were not enhanced with TCR stimulation for the siltuximab cohort. Together, these findings are consistent with an increase in IL-10 production promoting enhanced p-STAT3 signaling. Whether the increase in IL-10 and IL-27 produced by Teffs in the siltuximab-treated group contributed to the enhanced suppression by Tregs could not be addressed because of lack of sample availability. Overall, these findings demonstrated that siltuximab treatment led to alterations in CD4 T cell function distinct from those seen in tocilizumab-treated individuals. Whether the alteration in cytokine production and TCR-induced p-STAT3 contributed to the change in Treg-mediated suppression, or if other intrinsic changes in Teffs contributed, remains unclear.

A potential explanation for differential responsiveness to these drugs relates to dosing and pharmacokinetics. Siltuximab and tocilizumab have slightly different recommended weight-based dosing regimens, and the half-life of siltuximab in blood is approximately 10 days longer than that for tocilizumab. The pharmacokinetics of these drugs would affect their direct or indirect activities, especially with functional readouts at 14 days. Drug half-life and pharmacokinetics may also explain between-individual variability and the differential return to p-STAT3 responsiveness after tocilizumab exposure. Yet, the patterns of change in T cell responses seen with tocilizumab and siltuximab differed in direction, not solely magnitude, indicating that the differences were not due to pharmacokinetics alone. We propose that indirect effects on T cells via nonimmune and innate cell types may also help explain the differential response. Siltuximab and tocilizumab both have the capacity to block IL-6 responses via direct and trans signaling, albeit by binding different components of the IL-6/IL-6Rα complex. Yet, modest differences in the level of IL-6, IL-6Rα, and IL-6/IL-6Rα complexes driven by these therapies may influence the immune milieu. Intriguingly, IL-6 levels in serum increase after tocilizumab treatment in RA and after tocilizumab exposure in healthy individuals, likely due to receptor occupancy by the drug (41). Similarly, an agonistic effect can be seen when cytokine-specific antibody drugs bind their targets, which could be the case for siltuximab. Statistical modeling has suggested that, dependent upon dosing, shifting the equilibrium between free IL-6 and antibody/IL-6 complexes may change the rate of clearance of cytokine, prolonging its half-life and resulting in a paradoxical increase in IL-6–related measures (42). This may be of particular relevance to the indirect effects of IL-6 blockade on nonimmune and innate cell types that would differ from the effect of anti–IL-6R therapy.

Limitations of this study include the relatively small number of individuals studied and the short period of treatment. We acknowledge that the response to any therapy is heterogeneous and driven by genetic as well as environmental factors. One genetic factor that can influence the response to IL-6 is a common genetic variant in the IL-6R variant 358Ala (rs2228145 A>C). Individuals in this study were genotyped and as expected, IL-6R levels at day 0 strongly correlated with the IL-6R variant. However, we did not find any association with this variant and other outcome measures. We controlled for interindividual differences by enrolling a cohort of individuals into both studies. Notably, these individuals showed consistent drug-specific differences. Importantly, despite the limitations of interindividual variation, small sample size, and single-dose therapy, we observed significant changes in targeted T cell populations and detected differences between drug treatments.

In conclusion, we demonstrated, through the use of a single-dose intervention, how 2 therapies targeting the IL-6 pathway altered T cell responses in vivo in individuals with T1D. Our findings showed that monoclonal antibodies that target the receptor as compared with the cytokine differentially altered T cell responses, an observation that could not be made in vitro. Although this study was not designed to assess clinical outcome, its results raise the possibility that despite the failure of tocilizumab in a recent clinical trial to slow the rate of C-peptide decline in new onset diabetes, other IL-6–targeted therapies may be effective in T1D. Moreover, this finding may extend beyond IL-6–targeted therapies and suggests that as we move to target immune pathways therapeutically, the specific manner in which the pathway is targeted may affect outcomes. Short-term interventional studies may be a way to understand these differences and select therapies for clinical trials.

## Methods

### Study design.

The siltuximab and tocilizumab clinical studies were conducted independently and designed to assess predetermined immunological endpoints, not clinical outcomes. Both studies were single-arm, single-dose, and open-label. We enrolled 10 participants in the siltuximab study and 9 participants in the tocilizumab study. Note, an additional 3 participants were treated with tocilizumab after completion of sample collection for the primary cohort. Samples from these additional individuals were used to investigate effects on TCR-induced p-STAT3 signaling. All individuals were adults (18–45 years) and had T1D with disease duration of 4 months to 10 years after diagnosis, presence of at least 1 diabetes-related autoantibody, and detectable insulin secretion on a mixed-meal tolerance test at the time of the screening visit, which was within 60 days of study enrollment per standard protocols (43). HbA1c, C-peptide, and glucose values were measured using Clinical Laboratory Improvement Amendments–approved (CLIA-approved) protocols at Northwest Lipid Research Laboratories.

Abnormal results on complete blood counts (CBCs) at screening were exclusionary for both studies. CBC measurements were also conducted throughout each study; any clinically significant change triggered additional patient follow-up visits until resolution. Active viral infections (e.g., active Epstein-Barr virus or cytomegalovirus infection) or tuberculosis infection were exclusionary, as were recent vaccinations or exposures to biologic immunotherapies. CBC, viral load tests, C-reactive protein (CRP), QuantiFERON, standard (not high-sensitivity) CRP, and liver function tests were conducted using CLIA-approved protocols at the Virginia Mason Hospital Laboratory (Seattle, WA). Autoantibodies were measured by the Barbara Davis Center Antibody and HLA Core Facility using standardized radio-binding assays as previously described (44–46). Siltuximab was administered at 11 mg/kg, and tocilizumab was administered at 8 mg/kg, per dosing for each in FDA-approved settings. PBMCs and serum were collected at each visit for mechanistic assays and stored frozen for batch analysis at the end of each study. All samples were assayed in a blinded manner.

### Serum and plasma measurements.

Pharmacokinetic measurements of siltuximab levels in serum were performed by Janssen Pharmaceuticals. Tocilizumab levels in blood were measured using the Tocilizumab (Actemra) Pharmacokinetic ELISA (AffinityImmuno), per manufacturer instructions. Soluble IL-6R was measured in serum using the Human IL-6R Platinum ELISA kit (eBioscience).

### IL-6–induced p-STAT3 signaling, immunophenotyping, and intracellular cytokine stains.

There were 3 separate flow cytometry panels to assess cell-surface immunophenotyping, including IL-6R surface expression, IL-6–induced p-STAT3/p-STAT1 signaling, and intracellular staining of markers of Tregs and T helper subsets; antibodies are listed in Supplemental Table 2. Each panel included a LIVE/DEAD stain (Thermo Fisher Scientific). The cell-surface immunophenotyping and IL-6–induced p-STAT3/p-STAT1 signaling were performed as previously described (14). In brief, for the cell-surface immunophenotyping, unfixed cells were stained with antibodies against CD3, CD4, CD8, CD19, CD24, CD25, CD38, CD45RA, CD127, CCR5, CCR6, ADAM10, ADAM17, and IL-6R (Supplemental Table 2 and Supplemental Figure 2A). For the IL-6–induced p-STAT3 signaling, PBMCs were thawed, rested for 1 hour, and then stimulated with 2 ng/mL recombinant human IL-6 (Becton Dickinson) for 10 minutes at 37°C. Cells were then fixed and permeabilized using Fix buffer I and Perm buffer III (Becton Dickinson), respectively, and then stained with antibodies against CD3, CD4, CD8, CD25, CD27, CD33, CD45RA, CD56, CD127, p-STAT1 pY701, and p-STAT3 pY705 (Supplemental Table 2 and Supplemental Figure 4). The staining of mbIL-6R with either the UV4 or M5 monoclonal antibodies was carried out at room temperature for 20 minutes in the dark. In Supplemental Figure 3A, the mbIL-6R staining was done after preincubation with 200 μg/mL tocilizumab for 30 minutes at 37°C. In Supplemental Figure 3B, PBMCs were incubated with APC-labeled tocilizumab for 30 minutes at 37°C before the addition of PE-labeled IL-6R M5 monoclonal antibody for 20 minutes at 37°C; tocilizumab was labeled with APC using an Alexa Fluor Antibody Labeling kit (Invitrogen, A20186).

For intracellular staining, thawed and rested PBMCs were stimulated with PMA/ionomycin for 1 hour followed by an additional 3 hours in the presence of Brefeldin A. Cells were then stained with antibodies against CD3, CD4, CD8, CD45RA, CD161, CXCR5, FOXP3, Helios, ICOS, Ox40, PD-1, IFN-γ, IL-4, IL-17A, and IL-21 (Supplemental Table 2). CXCR5 failed to stain, so it was not included in the final analysis. In Figure 2, day 0 and day 14 were compared for all individuals except for 1 patient treated with tocilizumab where the pre-drug infusion sample was from screening (day –14) due to no remaining day 0 sample. Cells were acquired on an LSR III Fortessa flow cytometer (BD Biosciences) and data were analyzed using FlowJo version vX.06 or 10.7.1 (Tree Star).

### Treg-mediated suppression assay.

Teff resistance to suppression was measured using an in vitro Treg-mediated suppression assay using Teff cell surface expression of CD25 and CD134 as a surrogate marker of Treg-mediated suppression (6, 29). In brief, CD4^+^ T cells depleted of CD25^hi^ cells were isolated from PBMCs using a no-touch Miltenyi CD4 T Cell Isolation Kit II and positive Miltenyi CD25 microbeads II. CD4^+^CD25^+^CD127^lo^ Tregs from a single healthy donor were sorted, expanded, and frozen as described (47) and used a constant source of Tregs for all suppression assays. CD4^+^CD25^dim^ Teffs were cultured at 100,000 cells per well. Tregs were added at ratios of 1:4, 1:8, 1:16, and 1:32 (Treg/Teff), and Dynabeads CD3/CD28 T Cell Expander beads (Life Technologies) were added at a ratio of 1:28 (beads/Teffs). On day 2, Teffs were stained with CD25 and CD134 (Supplemental Table 2). For analysis, Teffs cultured in media alone were used to set gates for the various activation markers or proliferation. EF670 was used to identify Tregs. Percentage suppression (*s*) was calculated as follows: *s* = ([*a* − *b*]/*a*) × 100, where *a* is the percentage of CD25^+^CD134^+^ Teffs in the absence of Tregs and *b* is the percentage of CD25^+^CD134^+^ Teff cells in the presence of Tregs. Samples were collected on a BD Biosciences FACSCanto II, and data were analyzed using FlowJo V10.6.2.

### TCR-induced p-STAT signaling.

Total untouched CD3^+^ T cells were enriched from PBMCs using the human Pan T cell isolation kit (Miltenyi Biotec). Cells were stained with the Zombie Aqua Fixable Viability kit (BioLegend), rested for 1 hour in Immunocult serum-free and xeno-free XF T cell expansion medium (Stem Cell Technologies), and then activated with human CD3/CD28 T-Activator Dynabeads (Thermo Fisher Scientific) at a 1:28 bead/T cell ratio for 4 hours. Samples were stimulated in parallel with IL-6 (0.5 ng/mL, Thermo Fisher Scientific) and/or IL-10 (20 ng/mL, PeproTech) for 30 minutes. Immediately thereafter, cells were fixed with Cytofix/Cytoperm and permeabilized with Perm buffer III (BD Biosciences) overnight at –20°C. Samples were washed with 1× Perm/Wash buffer (BD Biosciences), blocked with human TruStain FcX FC receptor blocking solution (BioLegend), and then intracellular staining was performed in 1× Perm/Wash buffer for 45 minutes at room temperature. Total untouched CD4^+^ T cells were enriched from PBMCs using the Miltenyi CD4^+^ T cell isolation kit, rested, and then processed as above for intracellular staining of total STAT1 and total STAT5a or surface stained for cytokine receptor expression. Antibody panels are listed in Supplemental Table 2. In Figures 4 and 5 and Supplemental Figure 9, day 0 and day 14 were compared for all individuals except for 1 patient treated with siltuximab where the pre-drug infusion sample was from screening (day –14) due to no remaining day 0 sample. Cells were acquired on a BD Biosciences FACSCanto II and data were analyzed using FlowJo version vX.06 or 10.7.1 (Tree Star).

### Meso Scale Discovery assays for high-sensitivity detection of cytokines.

Custom U-PLEX panels of 8 assays/well (for siltuximab study) or 9 assays/well (for tocilizumab study) of a 96-well plate (referred to hereafter as 8- or 9-assay plates) were used to assess day 0 and day 14 T cell responses in 4-hour conditioned media from CD3/CD28 Dynabead-activated CD3^+^ T cells. Eight-assay plate analytes included IL-2, IL-6, IL-7, IL-10, IL-15, IL-21, IL-22, and IL-27. Nine-assay plate analytes included IL-6, IL-7, IL-10, IL-15, IL-21, IL-22, IL-27, TNF-α, and TNF-β. Activated cell culture supernatants were collected prior to processing cells for phospho-flow and stored at –80°C. Thawed samples were tested undiluted in duplicate, where unstimulated samples served as negative controls, and 24-hour T cell–conditioned media from a healthy control individual served as positive control. Calibrators and test samples were incubated overnight on coated plates and assayed according to the manufacturer’s instructions for U-PLEX Custom Biomarker Group 1 assays (human) (Meso Scale Discovery). Meso Scale Discovery assay plates were read on a Sector SQ120MM instrument. Data were analyzed using Meso Scale Discovery Workbench v4.0 software. Mean calculated concentration coefficient of variation was 4.10% and 2.95% for the 8-assay and 9-assay plate calibrators, respectively, demonstrating the high reproducibility of the U-PLEX assays, with limits of detection within the low to sub pg/mL range. Of note, IL-2 levels were above the calibrator curve fit range for nearly all siltuximab study samples, whereas IL-7 and IL-15 were below calibrator curve fit range; therefore, these analytes were not included in the comparison of the effect of siltuximab and tocilizumab on T cell production of cytokines.

### Statistics.

Statistical analyses included a Wilcoxon matched-pairs signed-rank test, Pearson correlation, and linear regression. Analyses were performed using GraphPad Prism v8.0 software and/or R (48). *P* values of less than 0.05 were considered significant.

### Study approval.

Both studies were approved by the Benaroya Research Institute’s IRB (siltuximab protocol IRB15085; tocilizumab protocol IRB15159). All participants provided written informed consent upon enrollment into the study. The siltuximab study was conducted under an FDA-approved Investigational New Drug (IND) application and was therefore registered on ClinicalTrials.gov (NCT02641522). The tocilizumab study was determined to be IND exempt, so it was not registered on ClinicalTrials.gov. Samples from healthy control individuals for Treg isolation were collected through Benaroya Research Institute’s Immune Mediated Disease Registry and Repository (protocol IRB07109).

## Author contributions

CS, GTN, CJG, and JHB conceptualized and designed the study. CS, SL, and CJG were responsible for enrollment, clinical study conduct, and clinical data collection. CH, MK, and BEJ measured IL-6R expression and phosphorylation of p-STAT3. MDM performed the ELISA to measure soluble IL-6. CH and MT performed the in vitro suppression assays. TH and MT performed the assays examining TCR-induced p-STAT3 signaling, including the multiplex assays measuring cytokine production. TH, KL, CH, MJD, SOS, and AH analyzed the flow cytometry data. CS, TH, KL, AMH, and JHB wrote the manuscript with assistance from all coauthors. CJG and JHB obtained funding and were responsible for the entire project.

## Supplementary Material

Supplemental data

## Figures and Tables

**Table 1:**
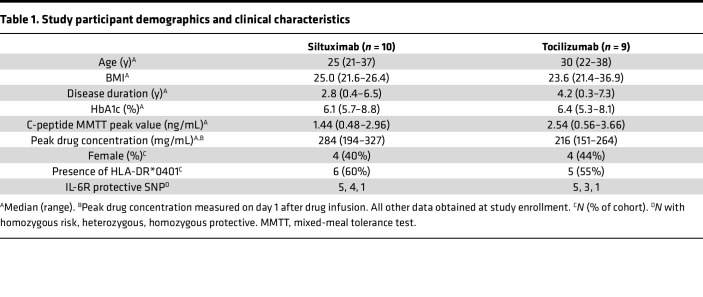
Study participant demographics and clinical characteristics

## Version 1 10/25/2022

In-Press Preview

## Version 2 11/22/2022

Electronic publication
