# Supplementary material for: IL-6–targeted therapies to block the cytokine or its receptor drive distinct alterations in T cell function
Source: JCI Insight. 2022 Nov 22;7(22):e159436. doi: 10.1172/jci.insight.159436 (PMC9746808; doi:10.1172/jci.insight.159436)
Supplement: Supplemental data [file jciinsight-7-159436-s178.pdf]

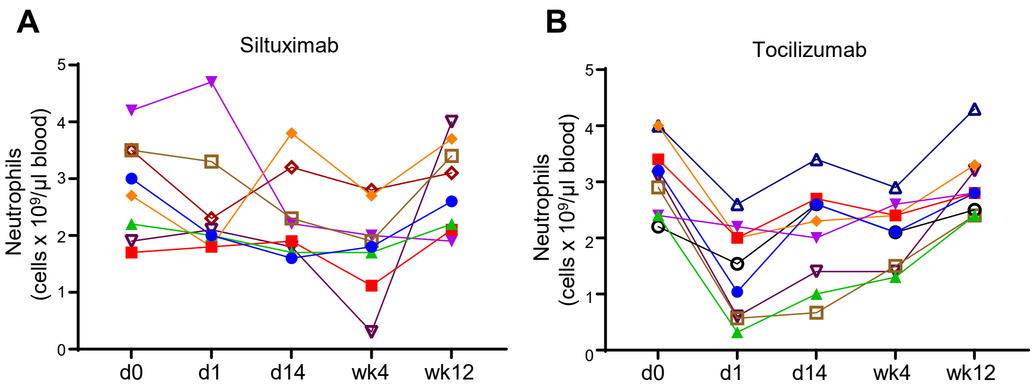

Supplemental Figure 1. Differential effects of siltuximab and tocilizumab on neutrophil counts. Neutrophil numbers were derived from complete blood counts (CBC) at each time point. Each line represents an individual subject; (**A**) siltuximab (n=10) and (**B**) tocilizumab (n=9).

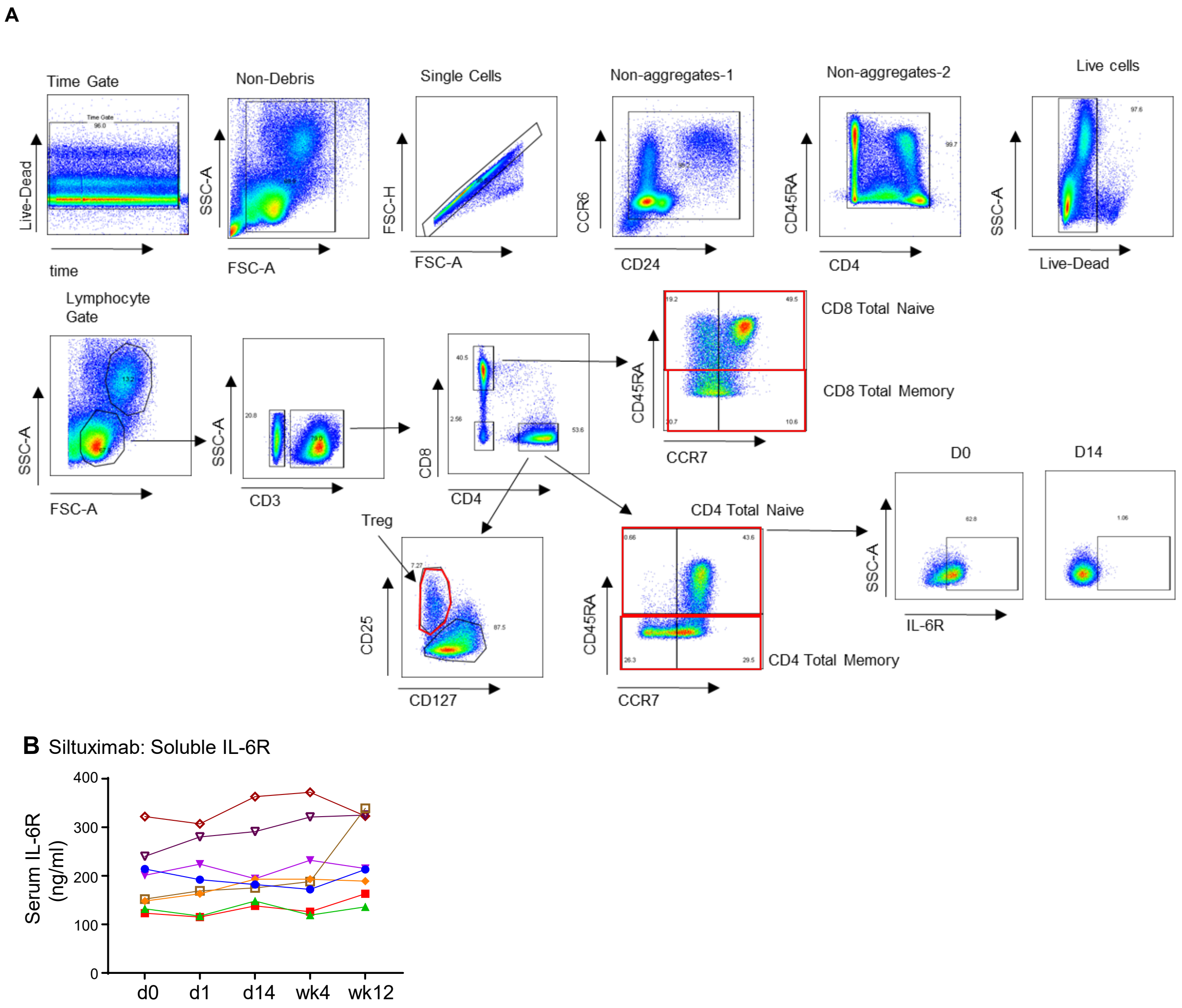

Supplemental Figure 2. Cell surface IL-6 receptor (IL-6R) gating strategy and soluble IL-6R serum levels (**A**) Cell surface IL-6R gating strategy (**B**) Serum levels of soluble IL-6R in the siltuximab cohort (n=8) measured by ELISA. Each line represents an individual subject.

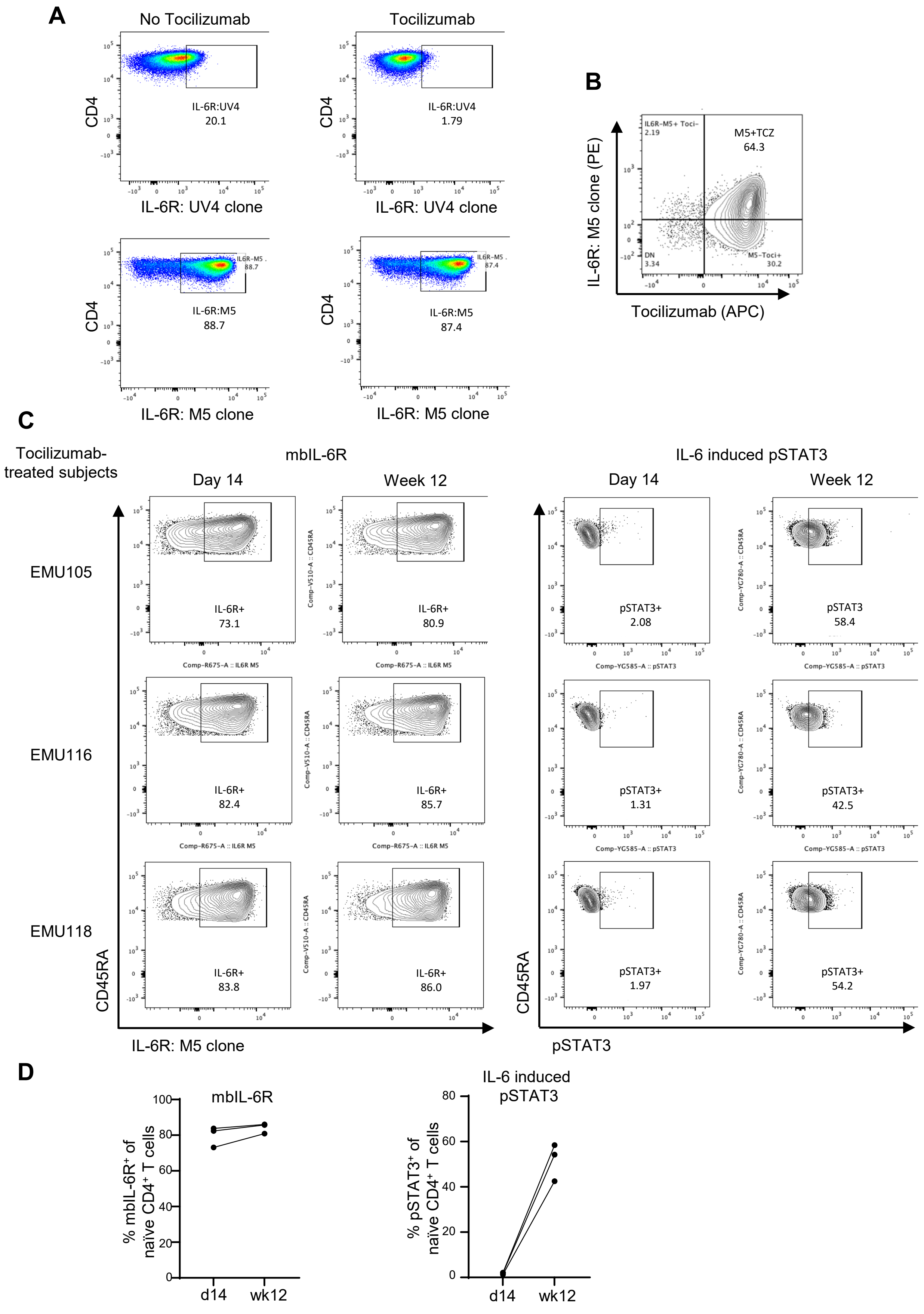

Supplemental Figure 3. mbIL-6R staining with UV4 and M5 monoclonal anti-IL-6R antibodies. (**A**) PBMC from a healthy control subjects cultured *in vitro* with or without tocilizumab (200µg/ml for 30 minutes) and then stained with UV4 or M5 monoclonal anti-IL-6R antibodies; gating shown for CD4^{+} T cells (**B**) Dual staining with APC-labeled tocilizumab and PE-labeled M5 monoclonal anti-IL-6R antibody on PBMC from healthy control subject; gating shown for naïve CD4^{+} T cells. (**C**) PBMC from three tocilizumab-treated individuals at day 14 and week 12 stained with M5 monoclonal anti-IL-6R antibody (*Left*) or pSTAT3 antibody (*Right*); gating shown for naïve CD4^{+} T cells. PBMC were stained with pSTAT3 antibody following stimulation with IL-6 (2ng/ml for 10 minutes) with gating shown for naïve CD4^{+} T cells. (**D**) Data from C quantified; frequency of mbIL-6R^{+} cells (*Left*) and pSTAT3^{+} cells (*Right*) in naïve CD4^{+} T cell compartment.

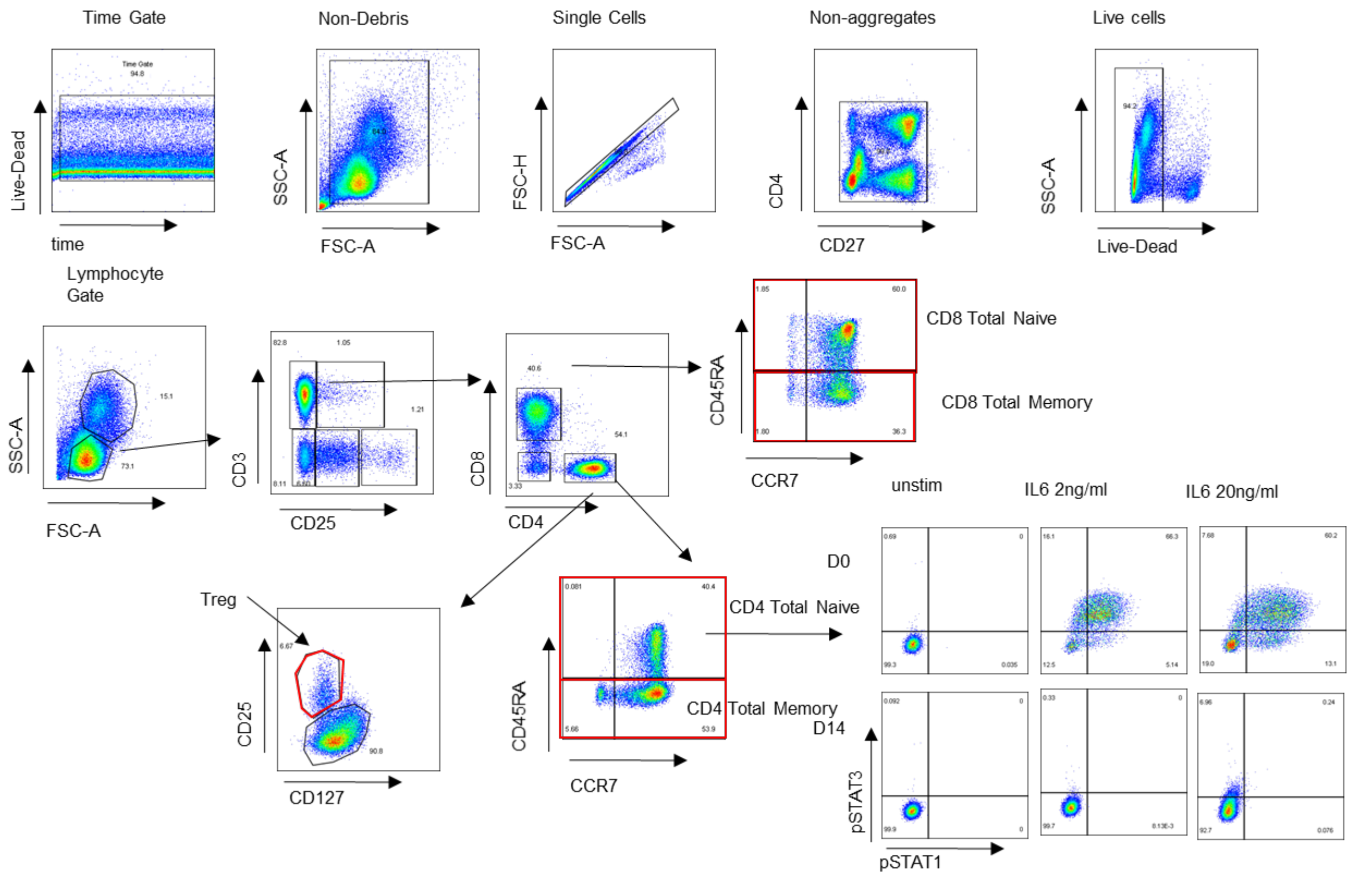

Supplemental Figure 4. IL-6-induced pSTAT1/3 gating

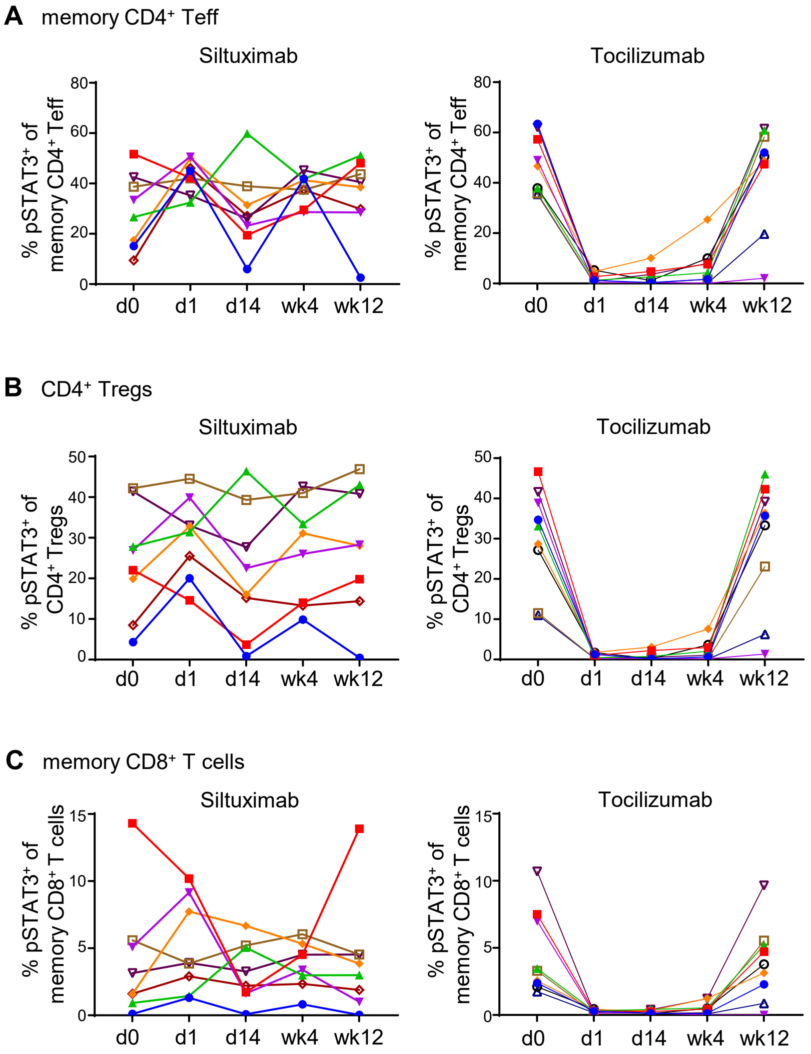

Supplemental Figure 5. Tocilizumab but not siltuximab suppresses IL-6-mediated phosphorylation of STAT3 in T cells. Thawed and rested PBMCs from siltuximab-treated or tocilizumab-treated T1D subjects were treated with recombinant IL-6 (2 ng/ml) for 10 minutes followed by staining for pSTAT3. Each line represents an individual subject; n=10 for siltuximab and n=9 for tocilizumab. Frequency of IL-6-induced pSTAT3-positive cells in the following T cell compartments (**A**) memory $CD4^+$ Teff, (**B**) $CD4^+$ Tregs, and (**C**) memory $CD8^+$ T cells.

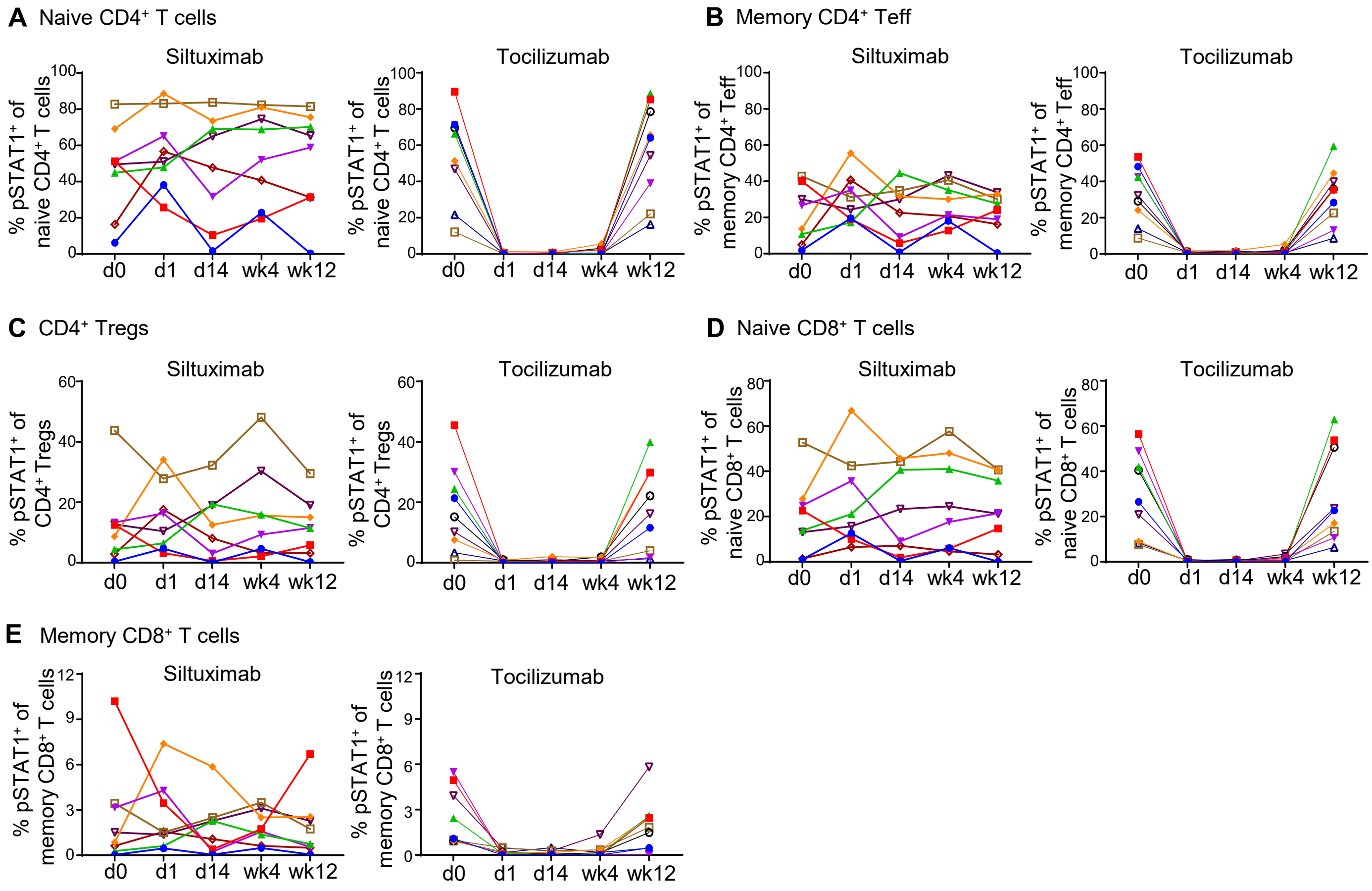

Supplemental Figure 6. Tocilizumab but not siltuximab suppresses IL-6-mediated phosphorylation of STAT1 in T cells. Thawed and rested PBMCs from siltuximab-treated or tocilizumab-treated T1D subjects were treated with recombinant IL-6 (2 ng/ml) for 10 minutes followed by staining for pSTAT1. Each line represents an individual subject; n=10 for siltuximab and n=9 for tocilizumab. Frequency of IL-6-induced pSTAT1$^+$ cells in the following T cell compartments: (**A**) naïve CD4$^+$ T cells (**B**) memory CD4$^+$ T cells, (**C**) CD4$^+$ Tregs, (**D**) naïve CD8$^+$ T cells and (**E**) memory CD8$^+$ T cells.

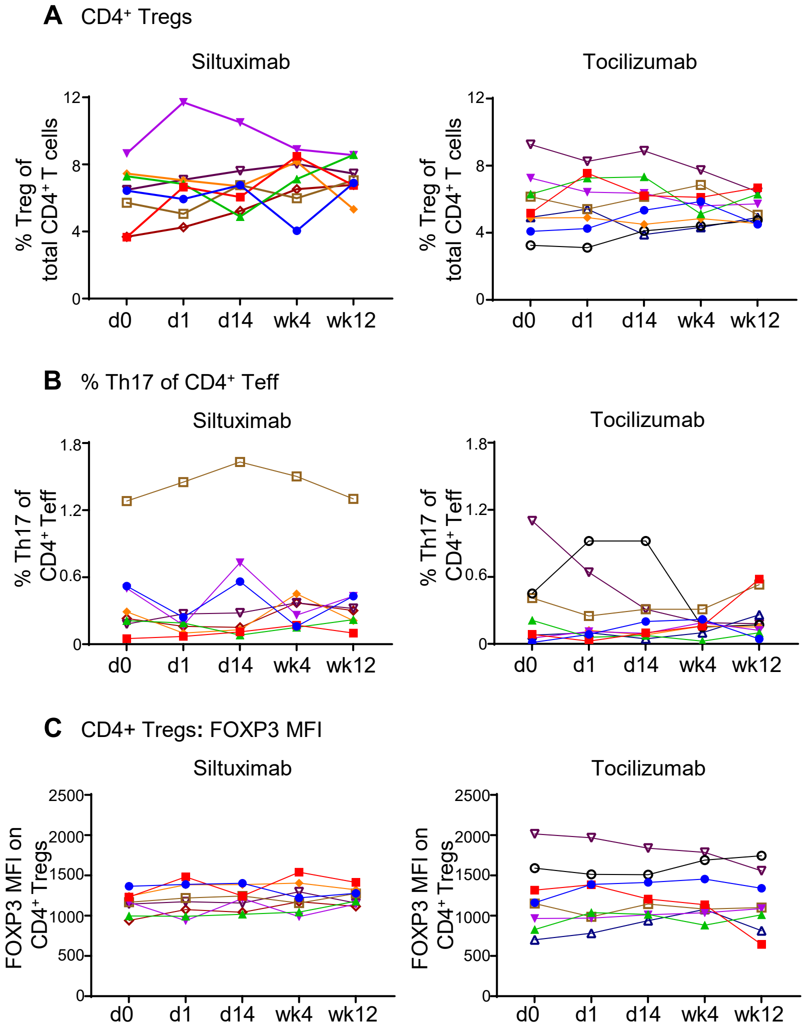

Supplemental Figure 7. Effect of siltuximab and tocilizumab on $CD4^+$ Tregs and Th17 cells. Each line represents an individual subject; n=8 for siltuximab and n=8 for tocilizumab except for a where n=9. (**A**) Frequency of Tregs ($CD4^+CD25^{hi}CD127^{lo}$) in $CD4^+$ T cell compartment. (**B**) Frequency of Th17 cells ($CD4^+FOXP3^-IL\text{-}17^+$) in $CD4^+$ Teff compartment. (**C**) FOXP3 mean fluorescence intensity (MFI) on $CD4^+$ Tregs

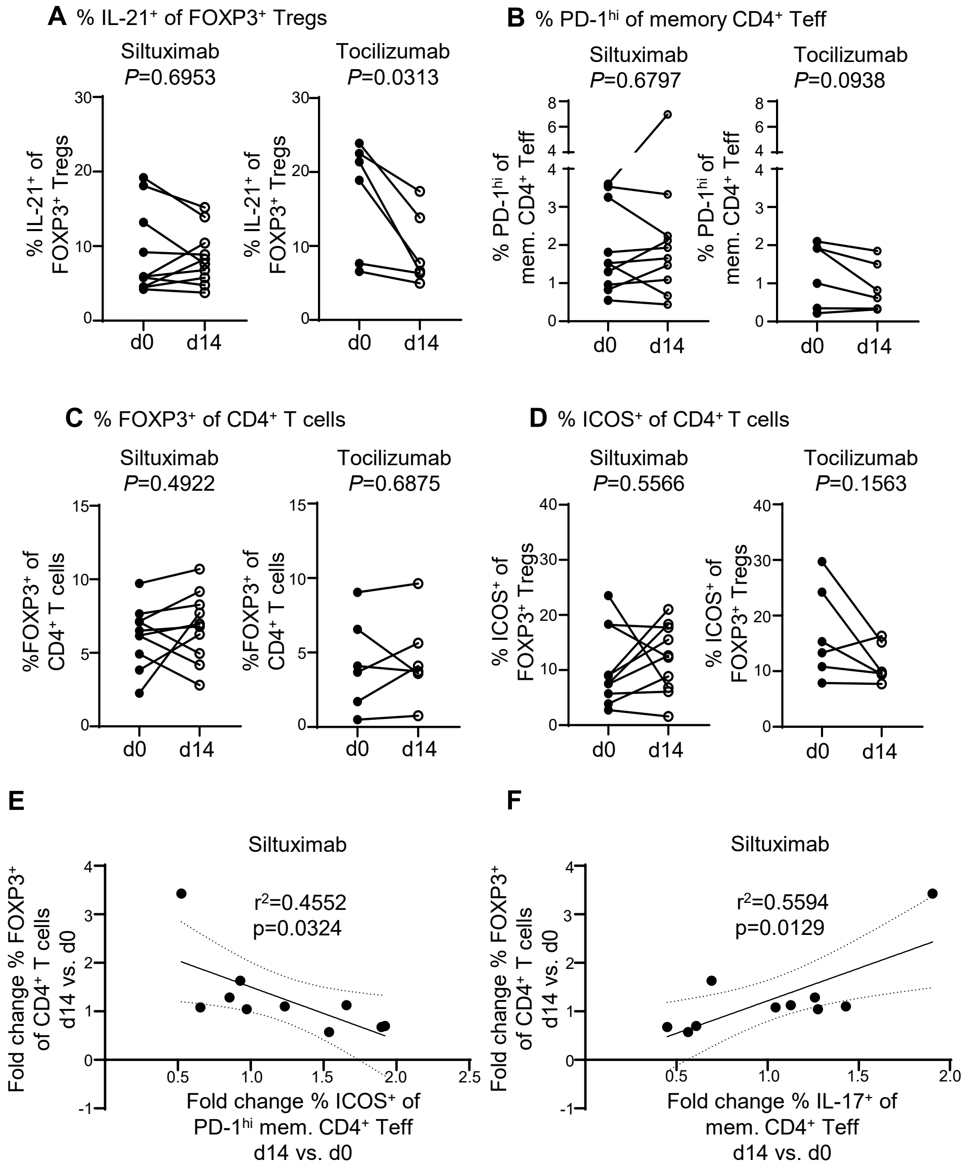

Supplemental Figure 8. Tocilizumab but not siltuximab decreases ICOS expression of T follicular helper cells. Thawed and rested PBMCs from siltuximab-treated or tocilizumab-treated T1D subjects were stimulated with PMA/ionomycin for 1 hour followed by an additional 3 hours in the presence of Brefeldin A. Each line represents an individual subject; n=10 for siltuximab and n=6 for tocilizumab. Solid circles represent day 0 prior to drug infusion (d0) and open circles represent day 14 post drug (d14). (**A**) Frequency of IL-21^{+} cells in FOXP^{+} Treg compartment. (**B**) Frequency of PD-1^{hi} cells in memory CD4^{+} T cell compartment. (**C**) Frequency of FOXP3^{+} cells in CD4^{+} T cell compartment. (**D**) Frequency of ICOS^{+} cells in FOXP^{+} Treg compartment. (**E**) Linear regression for siltuximab cohort showing negative correlation between fold change d14 versus d0 for frequency of ICOS^{+} cells in PD-1^{hi} memory CD4^{+} T cell compartment and fold change d14 versus d0 for frequency of FOXP3^{+} cells in memory CD4^{+} T cell compartment. (**F**) Linear regression for siltuximab cohort showing positive correlation between fold change d14 versus d0 for frequency of IL-17^{+} cells in memory CD4^{+} T cell compartment and fold change d14 versus d0 for frequency of FOXP3^{+} cells in memory CD4^{+} T cell compartment. Statistical tests: (**A**-**D)** Wilcoxon matched-pairs signed rank test; and (**E** and **F**) linear regression.

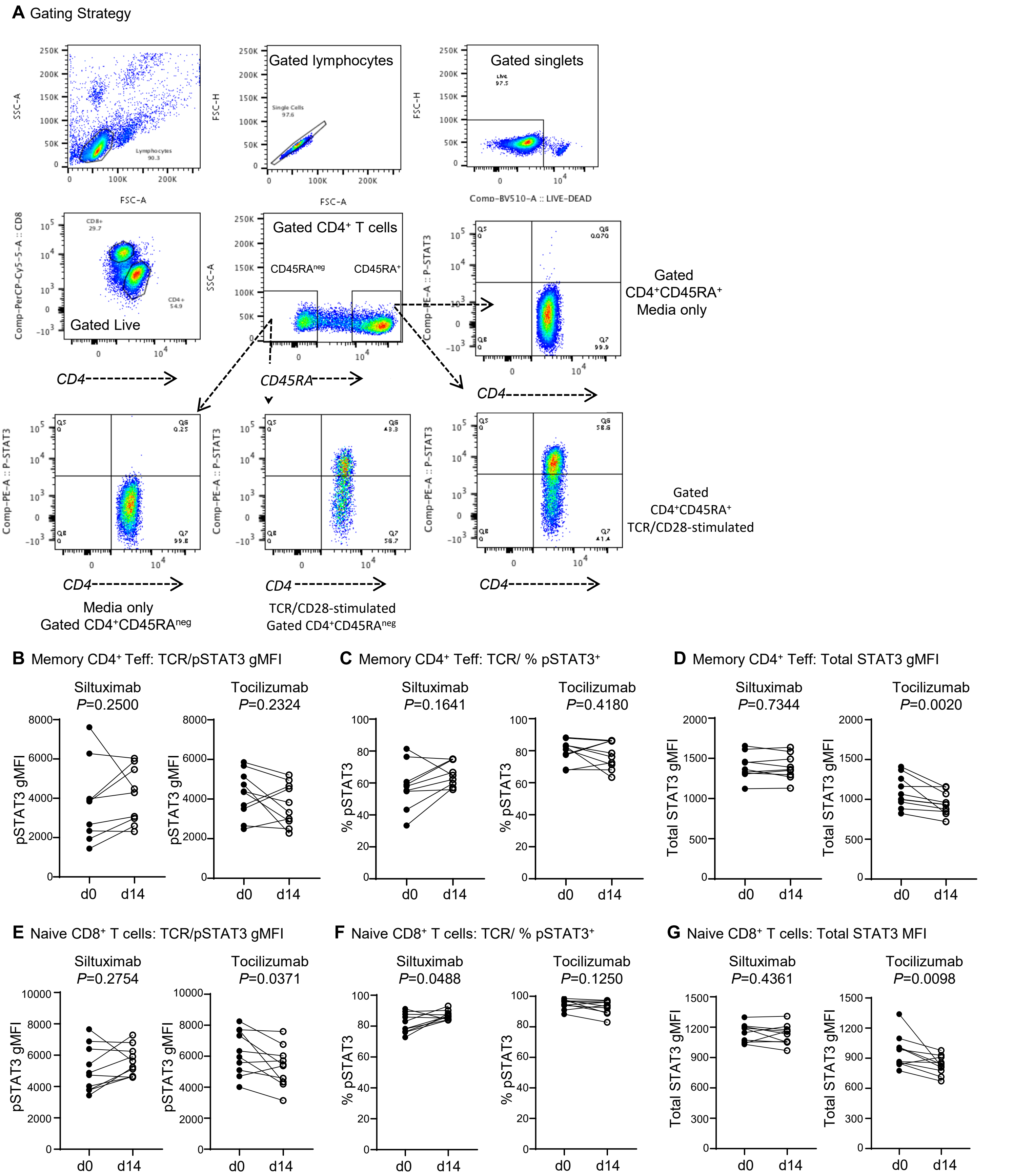

Supplemental Figure 9. Effects of siltuximab and tocilizumab on TCR-induced pSTAT3 signaling in memory CD4⁺ T cells and naïve CD8⁺ T cells. Enriched pan T cells from siltuximab-treated or tocilizumab-treated T1D subjects were stimulated or not with anti-CD3/anti-CD28 coated beads for four hours. Each line represents an individual subject; solid circles represent day 0 prior to administration of drug (d0) and open circles represent day 14 post drug (d14). (**A**) Gating strategy from a representative siltuximab-treated subject. (**B-D**) Memory $CD4^{+}CD45RA^{neg}$ T cells; siltuximab n=9, tocilizumab n=10. (**B**) pSTAT3 gMFI (**C**) Frequency of $pSTAT3^{+}$ cells (**D**) Total STAT3 MFI (**E-G**) Naive $CD8^{+}CD45RA^{+}$ T cells; siltuximab n=10, tocilizumab n=10. (**E**) pSTAT3 gMFI. (**F**) Frequency of $pSTAT3^{+}$ cells (**G**) Total STAT3 MFI. Statistical test: Wilcoxon matched-pairs signed rank test.

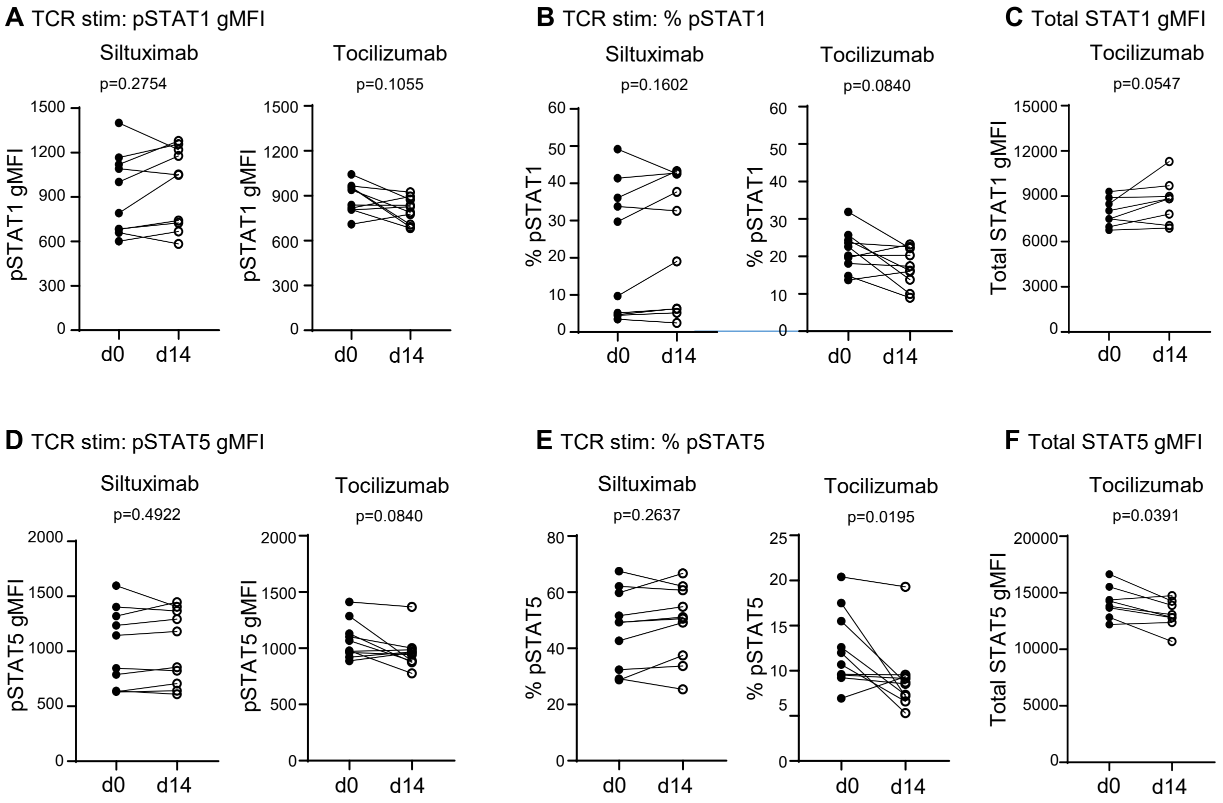

Supplemental Figure 10. Effects of siltuximab and tocilizumab on TCR-induced pSTAT1 and pSTAT5 signaling in naïve CD4+ T cells. (**A, B, D, E**) Enriched pan T cells from siltuximab-treated T1D subjects or tocilizumab-treated T1D subjects were stimulated or not with anti-CD3/anti-CD28 coated beads for four hours. Cells were stained for pSTAT1 and pSTAT5. (**C, F**) Enriched total $CD4^+$ T cells from tocilizumab-treated subjects were stained for total STAT1 and total STAT5. (**A-F**) Each line represents an individual subject; solid circles represent day 0 prior to administration of drug (d0) and open circles represent day 14 post drug (d14). (**A**) pSTAT1 gMFI of gated naïve $CD4^+$ T cells: siltuximab n=10, gated $CD4^+CD45RA^+$ T cells; tocilizumab n=10, gated $CD4^+CD27^+CD45RA^+$ T cells. (**B**) Frequency of $pSTAT1^+$ cells in the naïve $CD4^+$ T cell compartment; siltuximab n=10 and tocilizumab n=10. (**C**) Total STAT1 gMFI of unstimulated naïve $CD4^+$ T cells, n=8. (**D**) pSTAT5 MFI of gated naïve $CD4^+$ T cells; siltuximab n=10 and tocilizumab n=10. (**E**) Frequency of pSTAT5-positive cells in the naïve $CD4^+$ T cell compartment; siltuximab n=10 and tocilizumab n=10. (**F**) Total STAT5 MFI of unstimulated naïve $CD4^+$ T cells, n=8. Statistical test: Wilcoxon matched-pairs signed rank test.

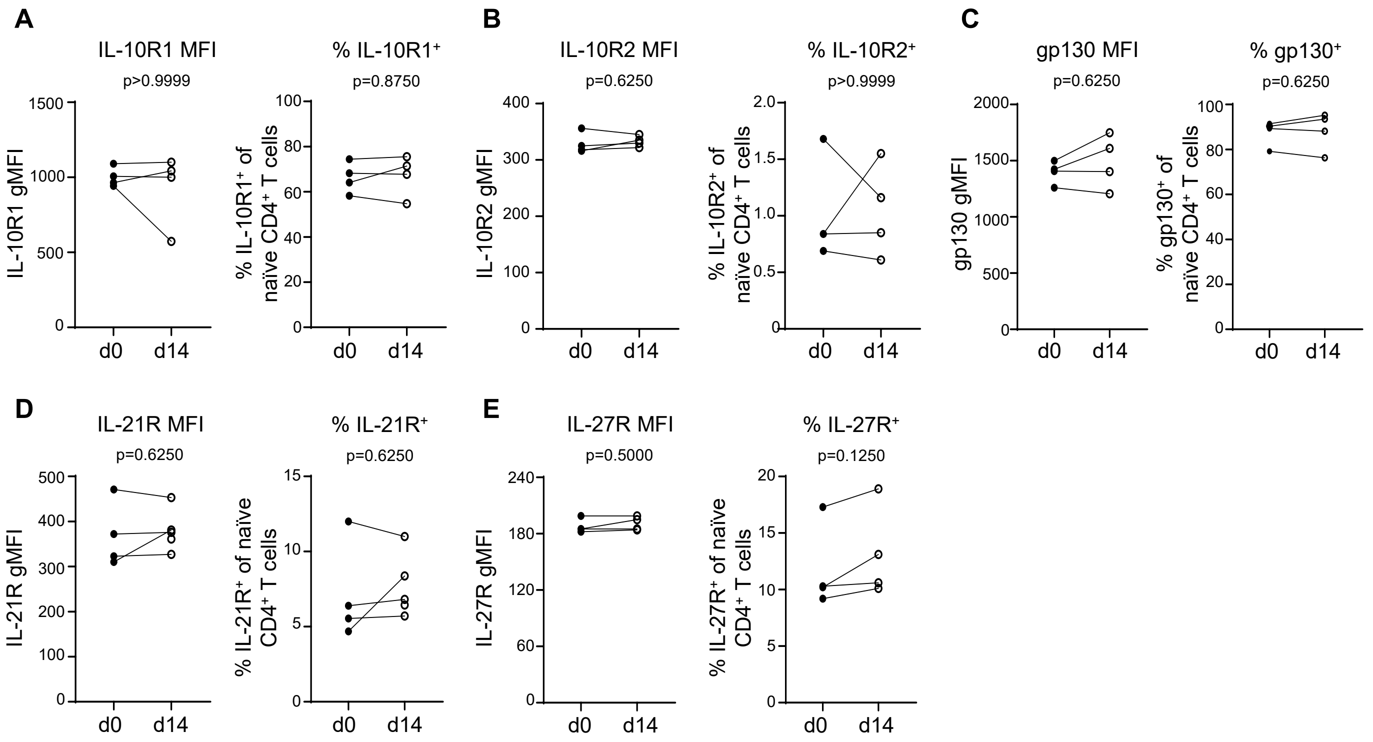

Supplemental Figure 11. Effect of siltuximab on cell surface expression of cytokine receptors. Enriched total CD4⁺ T cells from siltuximab-treated T1D subjects were stimulated with anti-CD3/anti-CD28 coated beads for four hours followed by cell surface staining for cytokine receptors. Each line represents an individual subject; solid circles represent day 0 prior to administration of drug (d0) and open circles represent day 14 post drug (d14). (**A**) *left,* IL-10R1 MFI (n=4); *right,* frequency of IL-10R1⁺ cells (n=4). (**B**) *left,* IL-10R2 MFI (n=4); *right,* frequency of IL-10R2⁺ cells (n=4). (**C**) *left,* gp130 MFI (n=4); *right,* frequency of gp130⁺ cells (n=4). (**D**) *left,* IL-21R MFI (n=4); *right,* frequency of IL-21R⁺ cells (n=4). (**E**) *left,* IL-27R MFI (n=4); *right,* frequency of IL-27R⁺ cells (n=4). Statistical test: Wilcoxon matched-pairs signed rank test.

Supplemental Table 1. Assays performed for each subject

	Subject	Sex	Age at Enrollment	Race	Ethnicity Hispanic / Latino	Figure 1	Figure 2	Figure 3	Figure 4	Figure 5	Ext. Data Figure 1	Ext. Data Figure 2	Ext. Data Figure 4	Ext. Data Figure 5	Ext. Data Figure 6	Ext. Data Figure 7	Ext. Data Figure 8	Ext. Data Figure 10	Ext. Data Figure 11	Ext. Data Figure 12
**Siltuximab**	EMU202[1]	Female	29	White, Caucasian	No	X	X	X	X	X	X	X	X	X	X	X	X	X	X	
	EMU204	Male	33	White, Caucasian	No	X	X	X	X	X	X	X	X	X	X	X		X	X	
	EMU205[2]	Male	37	White, Caucasian	No	X	X	X	X	X	X	X	X	X	X	X	X	X	X	
	EMU206	Male	21	Native Hawaiian, Other Pacific Islander	No	X	X	X	X	X	X	X	X	X	X	X		X	X	X
	EMU207[3]	Male	25	White, Caucasian	No	X	X	X	X	X	X	X	X	X	X	X	X	X	X	
	EMU209	Female	21	White, Caucasian	No		X	X	X	X						X		X	X	X
	EMU211[4]	Female	22	White, Caucasian	No	X	X	X	X		X	X	X	X	X	X	X	X	X	X
	EMU215	Female	21	White, Caucasian	No		X	X	X	X						X		X	X	
	EMU216	Male	25	White, Caucasian	No	X	X	X	X	X	X	X	X	X	X	X		X	X	X
	EMU217[5]	Male	37	White, Caucasian	No	X	X	X	X	X	X	X	X	X	X	X	X	X	X	X
**Tocilizumab**	EMU101[1]	Female	30	White, Caucasian	No	X	X	X	X	X	X		X	X	X	X	X	X	X	
	EMU103[2]	Female	22	White, Caucasian	No	X	X	X	X	X	X		X	X	X	X	X	X	X	
	EMU104[3]	Male	38	White, Caucasian	No	X		X	X	X	X		X	X	X		X	X	X	
	EMU105[4]	Male	26	White, Caucasian	No	X		X	X	X	X		X	X	X		X	X	X	
	EMU106[5]	Male	38	White, Caucasian	No	X	X	X			X		X	X	X	X	X			
	EMU107	Female	30	White, Caucasian	No	X		X	X	X	X		X	X	X			X	X	
	EMU109	Male	29	White, Caucasian	No	X	X	X			X		X	X	X	X				
	EMU110	Female	31	White, Caucasian	No	X	X	X	X	X	X		X	X	X	X		X	X	
	EMU111	Male	30	White, Caucasian	No	X	X	X	X	X	X		X	X	X	X		X	X	
	EMU116	Female	20	White, Caucasian	No				X	X								X	X	
	EMU118	Male	23	White, Caucasian	No				X	X								X	X	
	EMU119	Male	41	White, Caucasian	No				X	X										

[1-5] Overlapping subjects enrolled in both studies. Number pairs indicate same subject.

Supplemental Table 2. Flow cytometry antibodies used in the study

Assay	Target	Clone	Format	Vendor
**Phospho-flow: IL-6/pSTAT3, pSTAT1**	CD8	RPA-T8	BUV395	BD Biosciences
	CD27	L128	BV421	BD Biosciences
	CD4	SK3	eVolve605	Biolegend
	CD127	eBioRDR5	BV786	Biolegend
	CD33	P67.6	FITC	Biolegend
	CD3	UCHT1	PerCP-Cy5.5	Biolegend
	pSTAT3	4/pSTAT3	PE	BD Biosciences
	CD25	M-A251	PE-CF594	BD Biosciences
	CD56	NCAM16.2	PE-Cy7	BD Biosciences
	pSTAT1	4a	Ax647	BD Biosciences
	CD45RA	HI100	Alexa Fluor 700	BD Biosciences
	LIVE/DEAD FIXABLE Stain kit	N/A	Blue 488nm	Thermo Fisher Scientific
**IL-6 Receptor Expression**	CD38	HB7	BUV395	BD Biosciences
	CD8	SK1	BUV737	BD Biosciences
	CCR6	G0434E3	BV421	Biolegend
	CD24	ML5	BV510	Biolegend
	CD45RA	HI100	eVolve605	eBioscience
	CD4	SK3	eVolve655	eBioscience
	CD127	A019D5	BV786	Biolegend
	CCR5	3A9	BB515	BD Biosciences
	CD3	UCHT1	PerCP-Cy5.5	Biolegend
	ADAM17	1116333	PE	R&D Systems
	CD25	M-A251	PE-CF594	BD Biosciences
	IL-6R	UV4	PE-Cy7	Biolegend
	IL-6R	M5	PE	BD Biosciences
	ADAM10	SHM14	APC	Biolegend
	CD19	HIB19	AF700	Biolegend
	CCR7	G043H7	APC-Cy	Biolegend
	LIVE/DEAD FIXABLE Stain kit	N/A	Blue 488nm	Thermo Fisher Scientific
**Intracellular Staining**	IFNγ	B27	BUV395	BD Biosciences
	CD8	SK1	BUV737	BD Biosciences
	PD-1	EH12.2H7	BV421	Biolegend
	PD-1	EH12.2H7	BV785	Biolegend
	CD161	HP-3G10	BV510	Biolegend
	CD45RA	HI100	eVolve605	eBioscience
	CD4	SK3	eVolve655	eBioscience
	Ox40	ACT35	BV711	Biolegend
	CXCR5	J252D4	BV786	Biolegend
	CXCR5	RF8B2	BV421	BD Biosciences
	Helios	22F6	FITC	Biolegend
	IL-21	3A3-N2.1	PE	BD Biosciences
	FOXP3	259D/C7	PE-CF594	BD Biosciences
	ICOS	C398.4A	PE-Cy7	Biolegend
	IL-4	8D4-8	APC	BD Biosciences
	CD3	HIT3a	Alexa Fluor 700	Biolegend
	IL-17A	BL168	APC-Cy7	Biolegend
	LIVE/DEAD FIXABLE Stain kit	N/A	Blue 488nm	Thermo Fisher Scientific
**Suppression Assay**	CD25	M-A251	PE-Cy7	BD Biosciences
	CD3	UCHT1	BV421	Biolegend
	CD4	RPA-T4	APC-Cy7	Biolegend
	CD134	ACT35	PE	BD Biosciences
	Cell proliferation dye	N/A	eFlour670	eBioscience
	LIVE/DEAD FIXABLE Stain kit	N/A	Aqua 405nm	Thermo Fisher Scientific
**EMU2 Phospho-flow: TCR/pSTAT3, pSTAT1,pSTAT5**	pSTAT3	4/pSTAT3	PE	BD Biosciences
	CD45RA	HI100	BV421	Biolegend
	pSTAT5	47	Alexa fluor 488	BD Biosciences
	CD4	RPA-T4	APC-Cy7	Biolegend
	pSTAT1	4a	Alexa fluor 647	BD Biosciences
	CD8	RPA-T8	PerCP-Cy5.5	Biolegend
	CD25	M-A251	PE-Cy7	BD Biosciences
	CD5	UCHT2	BV605	BD Biosciences
	LIVE/DEAD FIXABLE Stain kit	N/A	Aqua 405nm	Thermo Fisher Scientific
**EMU1 Phospho-flow: TCR/pSTAT3, pSTAT5,pSTAT1, tSTAT3**	pSTAT3	4/pSTAT3	PE	BD Biosciences
**EMU2 Phosflow: unstimmed/pSTAT3, pSTAT5,pSTAT1, tSTAT3 (also did IL-10 stim as cytokine control)**	CD45RA	HI100	BV421	Biolegend
	pSTAT1	4a	Alexa fluor 488	BD Biosciences
	CD4	RPA-T4	APC-Cy7	Biolegend
	CD27	L128	BV711	BD Biosciences
	tSTAT3	M59-50	APC	BD Biosciences
	CD8	RPA-T8	PerCP-Cy5.5	Biolegend
	pSTAT5	47	PE-Cy7	BD Biosciences
	CD5	UCHT2	BV605	BD Biosciences
	LIVE/DEAD FIXABLE Stain kit	N/A	Aqua 405nm	Thermo Fisher Scientific

Supplemental Table 2 (cont.).

**EMU2: Surface stains Cytokine Receptor**	Il-10R/CD210	REAaffinity	PE	Miltenyi
	IL-27a	FAB	FITC/Vio-Bright515	R&D Systems
	CD4	RPA-T4	PerCP-Cy5.5	Biolegend
	IL-21a	17A12	PE-Cy7	Biolegend
	CD3	UCHT1	BV711	Biolegend
	gp130	2E1B02	PE/Dazzle	Biolegend
	CD45RA	HI100	BV421	Biolegend
	IL-10R2/CD210b	REA848	APC	Miltenyi
	LIVE/DEAD FIXABLE Stain kit	N/A	Aqua 405nm	Thermo Fisher Scientific
**EMU1: Intracellular Staining: tSTAT1,tSTAT5**	tSTAT5	REA549	PE	Miltenyi
	CD45RA	HI100	BV421	Biolegend
	CD27	L128	Alexa fluor 488	BD Biosciences
	CD4	RPA-T4	APC-Cy7	Biolegend
	tSTAT1	1/STAT1	Alexa fluor 647	BD Biosciences
	CD25	MA-251	PE-Cy7	BD Biosciences
	CD5	UCHT2	BV605	BD Biosciences
	LIVE/DEAD FIXABLE Stain kit	N/A	Aqua 405nm	Thermo Fisher Scientific
